# Global screening of health behaviors: Introducing Lev-screening (Lev-s)–development and psychometric evaluation

**DOI:** 10.1371/journal.pone.0315565

**Published:** 2024-12-26

**Authors:** Douglas Sjöwall, Felicia Stålhand, Greta Schettini, Petter Gustavsson, Tatja Hirvikoski

**Affiliations:** 1 Department of Women’s and Children’s Health, Pediatric Neuropsychiatry Unit, Center for Neurodevelopmental Disorders at Karolinska Institutet (KIND), Center for Psychiatry Research Karolinska Institutet, Stockholm, Sweden; 2 Habilitation and Health, Stockholm Health Care Services, Region Stockholm, Sweden; 3 Center for Neurodevelopmental Disorders at Karolinska Institutet (KIND), CAP Research Center, Region Stockholm, Sweden; 4 Division of Psychology, Department of Clinical Neuroscience, Karolinska Institutet, Stockholm, Sweden; 5 Independent Researcher; 6 Centre for Psychiatry Research, Department of Clinical Neuroscience, Karolinska Institutet, & Stockholm Health Care Services, Region Stockholm, Sweden; 7 Centre for Dependency Disorders, Stockholm Health Care Services, Region Stockholm, Stockholm, Sweden; Idaho State University, UNITED STATES OF AMERICA

## Abstract

Poor health behaviors have been identified as a critical factor for the burden on healthcare systems and individual suffering. However, comprehensive assessment of health behaviors is time-consuming and often neglected. To address this, we introduce the Lev-screening (Lev-s), a new, brief tool that covers multiple health behaviors. The Lev-s assesses ten health behaviors—physical activity, diet, alcohol use, tobacco use, illegal drug use, sleep, social relations, meaningful activities, sexual health, and screen health—using 33 items. This article details the development and psychometric evaluation of Lev-s with a sample of 2,279 participants aged 18–87. Test-retest reliability estimated as intra-class correlation coefficients for the different health behaviors ranged from .71 to .98 (n = 157), indicating moderate to excellent reliability. Lev-s showed associations with quality of life, demonstrated inter-correlations among included health behaviors, and detected group differences between individuals with and without neurodevelopmental conditions. The Lev-s exhibits satisfactory psychometric properties and holds promise as an efficient tool for screening of health behaviors.

## Introduction

Poor health behaviors (also called lifestyle behaviors and health-related behaviors) have been associated with negative short-term and long-term health outcomes, including early mortality. Therefore, the imperative for a paradigm shift towards prioritizing health behaviors within healthcare is compelling [1, 2]. A first step towards prioritizing health behaviors is to acknowledge them. While there are many in-depth assessments of important health behaviors, administering all that could be of relevance would take extensive time, and therefore risk not being done. To address this problem we introduce a new, brief, screening covering multiple health behaviors: Lev screening (Lev-s).

### From reactive to preventive healthcare

Non-communicable diseases are increasing worldwide and cause immense human suffering and strain on the healthcare systems [3, 4]. Non-communicable diseases are affected by health behaviors and a third of the overall world illness burden has been estimated to be associated with inadequate physical activity and poor diet alone [5]. Poor health behaviors are also strongly associated with mental health problems [6] demanding substantial healthcare resources [6, 7]. Addressing health behaviors is therefore important for preventing future mental and physical ill health, premature mortality as well as burden on the healthcare system [8].

### The need to acknowledge multiple health behaviors

While there are several instruments that cover health behaviors e.g., [9–11], there is to our knowledge no systematically evaluated screening instrument that cover more than a few areas. Our point of departure is that it is important to assess as many health behaviors as possible that can be important keys in promoting current and future psychological, social and physical well-being. Moreover, bringing awareness to multiple health behaviors could increase the likelihood of finding areas for which the patient’s intrinsic motivation is high enough for active participation in their health situation [12]. However, while including multiple health behaviors is important, the screening instrument needs to be relatively short to be feasible in outpatient contexts. A brief screening will also be more viable when patients are asked to complete it independently before meeting healthcare staff. The health behaviors included in Lev-s are described below under “construction”.

### Aim

The aim of this study is to, first, describe the systematic development and second, commence evaluating the psychometric properties of Lev-s.

## Method

The core principles of The Standards for Educational and Psychological Testing [13] was used to guide the development of the Lev-s. In line with our aim, first, the development of Lev-s is described to provide a rational for how we have decided on the specific items. Second, we describe the participants, materials and analyses that constitute the base for the psychometric evaluation.

### Development of Lev-s

#### Conceptualization

The purpose of creating Lev-s was to enable a first and fast screening of health behaviors in multiple healthcare contexts. To succeed with this, we strived to make Lev-s easy to use without the need for preparatory education or lengthy instructions. The health behaviors included should be relevant for adults, regardless of biological sex, gender identity, age, and mental or physical disabilities. Lev-s might be administered as self-rating or interview to bring awareness to health behaviors that impact current and future well-being. This is in line with a preventive healthcare focus and with integrating health behaviors to a larger extent in all healthcare processes [14]. While the items included aim to create awareness for both healthcare workers and patients of important aspects within each health behavior, it should ideally be complemented with a more in-depth assessment, if the purpose goes beyond detecting poor health behaviors.

#### Construction

Lev-s was developed in an outpatient habilitation/disability health care organization. The choice of included health behaviors was guided by their potential to affect mental and physical health. The following ten health behaviors were included: physical activity [15], diet [16], alcohol [17] tobacco, [18], illegal drugs [19], sleep [20], social relations [21], meaningful activities [22], sexual health [23] and screen health [24]. To decide on what items to include we reviewed the literature and consulted experts for each health behavior as well as healthcare workers from different disciplines. Typically, we identified important aspects to acknowledge reviewing the literature. We then presented items to experts and healthcare workers for each health behavior to get feedback on if the aspects covered were relevant and if items were formulated and scored in accordance with what they perceived as correct. Experts consulted included senior researchers specializing in various health behaviors. The healthcare professionals involved were psychologists, physiotherapists, occupational therapists, dietitians, and social workers, all with extensive experience in working with individuals representing a wide range of cognitive and physical abilities.

#### Rationale for health behaviors and items in Lev-s

We aimed to create a screening instrument with a broad applicability including individuals with challenges in mental and physical functioning. To ensure the relevance of each health behavior and identify what items to include we reviewed the literature regarding its *importance* for health, *frequency* in the general population and in individuals with neurodevelopmental conditions, and *central aspects* for items to cover. The choice of what aspects to include within each health behavior was based on what the scientific literature suggested to be important for an individual’s current and future well-being. We strived to included mostly objective aspects when possible (e.g., frequency of sedentary behavior, quantity of alcohol, tobacco) but for a few health behaviors the scientific literature implied that a more adequate representation of that health behavior should also include subjective aspects (e.g., satisfaction with sleep, social relations and sex) and functioning (e.g., pain, dysregulation, displacement). All health behaviors and central aspects within each health behavior included in the Lev-s are displayed in Table 1 and motivated below.

**Table 1:** Health behaviors and their central aspects.

Health behaviors	Central aspects included
Physical activity	Physical activity vigorous intensity
	Physical activity moderate intensity
	Sedentary behavior
	Interruption of sedentary behavior
Diet	Nutrition
	Regularity
	Eating behaviors
Alcohol	Frequency
	Quantity
	Frequency excessive quantity
Tobacco	Presence
	Frequency
Illegal Drugs	Presence
	Frequency
Sleep	Duration
	Continuity
	Regularity
	Alertness
	Satisfaction
Social relations	Quality
	Loneliness
	Satisfaction
	Quantity
Meaningful activities	Frequency leisure activities
	Satisfaction meaningful occupation
	Excessive frequency
Sexual health	Satisfaction
	Impairment
	Safety/trauma
Screen health	Excessive frequency
	Dysregulation
	Behavioral displacement
	Avoidance

Results for the identified central aspects within each health behaviors included in Lev-s.

*Physical activities*. *Importance*: Physical inactivity is a big risk factor for early mortality by increasing the risk for non-communicable diseases [25]. Physical activity affects mental health symptoms for the majority population as well as individuals with mental health problems or chronic diseases [26]. *Frequency*: More than 80% of adolescents and 25% of adults do not reach recommended levels of physical activity [25]. Having a neurodevelopmental condition can be associated with both lower [27–29] and higher [30] physical activity levels. *Central aspects*: The international recommendations regarding physical activity for adults 18–64 years, with or without disabilities, are 150–300 minutes of moderate-, or 75–150 minutes of vigorous-intensity aerobic physical activity per week. Moreover, limiting sedentary time with any kind of physical activity is recommended [31].

*Diet*. *Importance*: A unhealthy diet is related to non-communicable diseases [32] such as diabetes and mental health problems [33]. *Frequency*: Unhealthy eating is the single most important risk factor in terms of the total burden of disease globally [34]. Close to 60% of the European population are overweight or obese [35] and 1–4%, depending on gender, suffer from eating disorders [36]. Having a neurodevelopmental condition is associated with elevated risk both for eating disorders [37] and obesity [38, 39]. *Central aspects*: There is a wide international agreement on what constitutes a healthy diet. Recommendations suggest a regular food intake largely based on vegetables, root vegetables, legumes, fruits, berries, nuts, seeds, whole grains, fish, seafood, vegetable oils, as well as lean and unsweetened dairy products [40]. Another aspect of importance is eating behavior. This may include eating to ease depressive states or anxiety as well as when eating because of exhilaration [41, 42].

*Alcohol*. *Importance*: Consumption of alcohol increases the risk of cancer, cardiovascular disease, sleep difficulties, mental health problems, violence-related injuries, economic vulnerability, exclusion, and ultimately mortality [43]. *Frequency*: Alcohol cause around 3 million deaths globally per year and is attributable to around 5% of the total burden of disease [43]. Problematic use is elevated in neurodevelopmental conditions [44, 45]. *Central aspects*: Control over drinking and impact on daily life are important factors for alcohol use disorders. However, we found that there was substantial evidence to focus on frequency and quantity to bring attention to that high consumption without a current negative impact is strongly associated with future health problems [46].

*Tobacco*. *Importance*: Tobacco use has a negative effect on most of the body’s organ systems and lead to premature mortality in up to half of users who do not quit [47]. *Frequency*: Worldwide, about 20% use tobacco and smoking causes around 8 million deaths yearly [47]. High levels of Attention-Deficit/Hyperactivity Disorder (ADHD) symptoms is a risk factor for tobacco use [48] while autistic individuals report less use than non-autistic counterparts [49]. *Central aspects*: Since there are numerous health benefits of quitting tobacco use, the general recommendation is to abstain from it completely [47]. However, as there is a dose-response relation between tobacco use and health [50] it is important to include not only presence of use but also the frequency.

*Illegal drugs*. *Importance*: Using illegal drugs, (e.g., cannabis, amphetamines, opiates, hallucinogens) or misuse of prescription drugs is associated with both short-term and long-term health risks [51]. Risks vary depending on substance and individual fragility, but some examples of short-term risks are increased heart rate, blood pressure, and irritability. Examples of long-term risks are drug induced psychosis, changes to brain function such as memory loss, and depression [51]. *Frequency*: The European Drug Report 2023 indicates a rise in the availability and consumption of various illegal drugs, including cannabis, cocaine, and synthetic drugs. Individuals with ADHD and autism are more likely to use illegal drugs as compared to neurotypicals [44, 52]. *Central aspects*: To detect a possible use of illegal substances, we included items for “presence” and “frequency” of illegal substances and misuse of prescription drugs [53].

*Sleep*. *Importance*: Enough sleep is vital for mental and physical health. Short and long-term consequences of poor sleep include depression, stress, anxiety, impaired cognitive functioning, and non-communicable diseases [54]. *Frequency*: The prevalence of insomnia is estimated to be 10%–30% worldwide but the incidence of sleep-related problems are even higher in individuals with neurodevelopmental conditions [55]. *Central aspects*: Importantly, neither too little nor too much sleep is healthy [56]. Beyond duration, several qualitative aspects of sleep affect health such as poor sleep efficiency, i.e. many awakenings and/or difficulty falling asleep [57] and sleep regularity, i.e. a similar sleep pattern every day. Furthermore, the ability to maintain attentive wakefulness, and subjective sleep satisfaction are factors that are associated with well-being [58].

*Social relations*. *Importance*: Close relationships affect physical and mental health [59]. Relatedly, loneliness is associated with several health aspects such dementia and mental health problems [60] and non-communicable diseases [61]. *Frequency*: About a third of the general population report feeling lonely and about a fifth experience social challenges with elevated risk for individuals with neurodevelopmental conditions [62]. *Central aspects*: How much a person has the sense of trust in close relations and that they will receive support when help is needed, is important for social well-being. The desire for social relationships can differ among individuals, therefore the subjective feeling of satisfaction with social relations is important to consider [63]. Still there are indications that individuals with few friends often have a desire to have more [64] and we received the suggestion from experts on neurodevelopmental conditions to also assess how many friends one has offline and outside the family.

*Meaningful activities*. *Importance*: Doing something one finds meaningful, including occupation and leisure activities, gives a sense of restoration, identity and well-being [22]. A lack of meaningful activities is related to reduced mental and physical health [65–67]. *Frequency*: Due to the large variation in contexts and definitions for meaningful activities [68] it is difficult to provide an estimation of frequency of dissatisfaction with activities and if individuals with neurodevelopmental conditions are better or worse off than neurotypical peers. However, individuals with disabilities, disease or old age may be challenged with physical, cognitive, and social barriers that limit meaningful activities [66, 68]. *Central aspects*: As mentioned above, central aspects to assess should include both enjoyable free-time interests and the more general aspect of feeling that one has meaningful activities in everyday life [22]. It is also important to acknowledge that meaningful activities, when performed extensively, can be unhealthy by limiting other important health behaviors [69].

*Sexual health*. *Importance*: Sexual health affect mental, social and physical well-being [70]. *Frequency*: About half of both the middle aged sexual majority and minority populations are not satisfied with their sexual health [71]. Individuals with neurodevelopmental conditions are less satisfied than neurotypical individuals, and at higher risk of being victimized and engage in more risky behaviors [72]. *Central aspects*: The perceived importance of sex varies between individuals. Important aspects of sexual health are connected to *satisfaction* (e.g., informed choices, and action in relation to sexuality), and *safety* (e.g., coercion, discrimination, or violence). Yet another aspect is *functional impairments* such as pain, diseases or physical obstacles limiting the ability to have sex [23].

*Screen health*. *Importance*: Excessive screen time is associated with poor diet, depression, and reduced quality of life [24, 73]. Screen-use may also influence other health behaviors such as sleep [74] and physical activity [75]. *Frequency*: Screentime is rising at all ages [76] and there is an association between mental health problems and excessive screen use [77]. Although there are indications of a higher screen time in children with neurodevelopmental conditions, this effect is less studied and more ambiguous in adults [78]. *Central aspects*: Based on expert suggestions we included the following aspects to detect problematic screen health: 1) duration, 2) difficulties regulating use, 3) impact on other health behaviors 4) screen time as means of escape from reality.

#### Scoring

**Fig 1:**
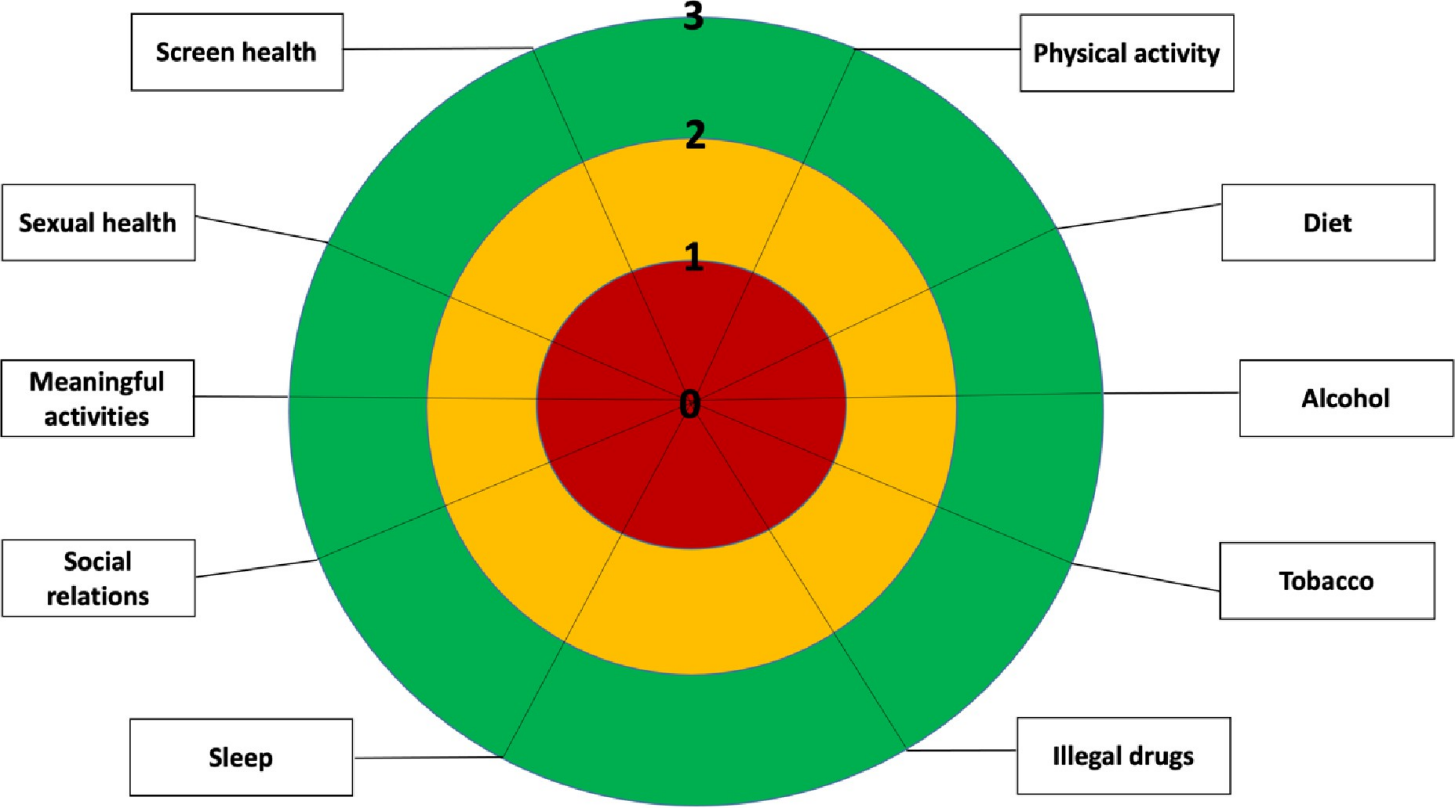
An overview diagram consisting of three-color fields: The inner field is red, symbolizing at high risk for ill health/high gain if changed; the middle yellow field symbolizes at risk for ill health; and the outer green field symbolizes healthy habits. Surrounding the figure are the 10 different health behaviors included in Lev-s. Markings can be made on the figure to visualize the score for each health behavior.

We used a quasi-absolute scale [79] where each response option is clearly defined. What is healthy or unhealthy does not change with population or context but on the current scientific understanding. Since the purpose of the instrument is to assess health behaviors, it was considered that an individual scoring low across many health behaviors might feel discouraged. To mitigate this, the instructions to read before using Lev-s included motivational messages such as how to manage disappointment and to see the potential to feel better, applicable both for self- and interview administration. Items are scored on a 4-point scale (0–3) with a few exceptions. Exceptions were made when it made sense acknowledging any level of difficulty as “high risk” to encouraging further discussions/investigations for the purpose reducing the problem. As an example, together with experts in sexual health we reasoned that it did not make sense grading answers on a 4-point scale for the item *Is sex difficult for you*: *do you experience pain*, *have a disease*, *or any other barrier that affects your ability to have sex*? Instead, we included the following response alternatives: *Yes*, *my ability to have sex is negatively affected by this* (0 points), *Yes*, *but I am satisfied with it nonetheless* (3 points), and *No*, *my ability to have sex is not hindered by this* (3 points). The score for each health behavior can be entered in circular figure with the traffic light analogy to provide an overview (see Fig 1). Importantly, the figure exhibit not just the risk areas but also relative strengths. Building on relative strengths, such as healthy social relations, can be important when addressing other health behaviors [80].

Each health behavior was assessed using 1–6 items, tailored to capture various aspects of the behavior (Table 1). The total score for each health behavior was determined either by averaging the item scores or by using the minimum score, depending on which method more accurately reflected the behavior in question. For "Diet", items covered both nutritional intake and eating behaviors. Given the diversity of items, using the average score could obscure harmful eating behaviors. For instance, a participant might score high on nutritional items (indicating good nutrition) but low on eating behavior items (indicating harmful behaviors such as overeating or undereating). In such cases, averaging the scores would fail to highlight these harmful behaviors (see S1 File for a detailed description of how the total scores are obtained for each health behavior). As part of the development, inter-rater reliability was investigated and found to be excellent (0.98–0.99) for all health behaviors in a separate study including last year students from different healthcare professions. The students also gave suggestions on the need for clarifications that were included in the updated version [81].

### Psychometric analyses of Lev-s

#### Participants and procedure

Recruitment to participate in this online self-rating study was done through social media (mainly Facebook and Instagram) and interest organizations for various disabilities in Sweden in 2022-10-31-2023-05-30. Inclusion criteria were being 18 years or older and Swedish speaking. Two samples were used to investigate the psychometric properties of Lev-s. Participants were informed about the purpose and what organization that conducted the study. They were informed of that they participated anonymously and that they could end participation at any time. Hence, no written consent was gathered. No compensations were given for participation. The study was approved by the Regional Ethics Committee in Stockholm (2022-02920-01).

The first recruitment campaign was directed to the general population with the instruction to compete the questionnaires, including Lev-s, twice two weeks apart. The instruction was to note in in their calendar to revisit the link to the online study 2 weeks after completing Lev-s the first time. Participants entered if it was the first or second time completing the self-assessment. If it was the second entry, they were asked to describe possible changes to the health behaviors that had occurred since the first time they answered the same questions. Cases where changes had occurred were excluded as the purpose was to examine consistency in ratings between time points under the assumption that no major change in health behaviors have occurred (i.e., test-retest reliability). Twenty percent of the total sample completed the test-retest within 2–4 weeks. Participants were anonymous but paring the data from the two time points was easily done based on their unique response pattern for the background questions, time of entry, and if it was the first or second time completing the self-assessment (see Table 2 for demographic of this sample).

**Table 2:** Participant characteristics for test-retest sample.

	n = 157
**Mean age (SD) (range)**	41(11) (19–64)
**Gender female *n* (%)**	139 (89%)
**Highest education *n* (%)**	
9-year compulsory school or less	2 (1%)
High school	39 (25%
University degree or higher	116 (74%)
**Occupation *n* (%)**	
Employed/student part or fulltime	145 (92%)
Unemployed/sick leave/disability pension part or fulltime	12 (8%)
**Living situation *n* (%)**	
Alone	49 (31%)
With partner and/or children	104 (66%)
Other	4 (3%)
**Self-reported mental and physical health problems** ^**a**^	
Neurodevelopmental conditions	16 (10%)
Anxiety and/or depression	29 (19%)
Physical illness/diseases	78 (50%)
No mental or physical health problems	60 (38%)

^a^ Participants may belong to multiple sub-categories

In the second recruitment campaign the purpose was to sample individuals with disabilities. These sample were combined with the sample from the first recruitment to increase variation in the data and to enable comparison based on disability (see Table 3 for demographic of the combined sample). Participants were anonymous and thus we did not request written consent. However, all participants were given detailed information online about what type of questions that were included in the study and that they could end participation at any time.

**Table 3:** Participant characteristics for the lager sample.

	n = 2279
**Mean age (SD) (range)**	44 (12) (18–87)
**Gender female *n* (%)**	1848 (81%)
**Highest education *n* (%)**	
9-year compulsory school or less	91 (4%)
High school	617 (28%
University degree or higher	1500 (68%)
**Occupation *n* (%)**	
Employed/student part or fulltime	2088 (86%)
Unemployed/sick leave/disability pension part or fulltime	350 (14%)
**Living situation *n* (%)**	
Alone	610 (27%)
With partner and/or children	1548 (68%)
Other	109 (5%)
Self-reported mental and physical health problems*	
Neurodevelopmental conditions	411 (19%)
Anxiety and/or depression	561 (26%)
Movement disability	180 (8%)
Physical illness/diseases	1093 (49%)
No mental or physical health problems	1146 (51%)

^a^ Participants may belong to multiple sub-categories

#### Materials

All participants completed questionnaires for demographic data, health behaviors and quality of life. Demographic information, diagnoses, and treatments were self-assessed using the Current Life Situation Form [82]. The Lev-s includes ten health behaviors, 33 items covering the aspects described in Table 1. A more detailed description of Lev-s is provided above under the heading “Scoring”. The English translation of the items included in Lev-s along with instruction for how to administer it as an interview as well as the scoring instruction can be found as supporting information (S1 File). An aspect of validity would be to compare Lev-s with well-established screening instruments specific to each health behavior. However, including numerous questionnaires in this study presents a challenge, reflecting the very issue we seek to address with the development of a streamlined, comprehensive screening tool for health behaviors. Instead, as a first step to address validity, the WHOQOL-BREF was included to provide an assessment of participants’ quality of life alongside specific health behaviors. The WHOQOL-BREF consists of 26 items encompassing four domains of quality of life: physical, psychological, social, and environmental. Cronbach’s alpha (n = 2241–2260), calculated for the baseline assessment) for the physical domain = .72, psychological = .87, social = .65, environmental = .83. The low Cronbach’s alpha for the social domain is in line with a previous study including a sample with a similar age range [83]. Removing item “How satisfied are you with your sex life?” increased Cronbach’s alpha to .72. We report analyses including the social domain with and without this item included.

#### Analyses

First, test-retest reliability of the Lev-s was investigated with ICC using SPSS Version 28. Scores from two testing occasions (T1 and T2) were collected for each participant. We used a two-way mixed-effects model focusing on the absolute agreement of individual scores across the two testing times, using the single measure ICC statistic. This approach evaluates the degree to which each individual’s average score at T1 is identical to their score at T2. When interpreting ICC values for reliability, the following ranges are used: less than 0.5 indicates poor reliability, between 0.5 and 0.75 indicates moderate reliability, between 0.75 and 0.9 indicates good reliability, and greater than 0.90 indicates excellent reliability [84].

Second, Pearson’s correlations coefficients were used to assess associations to quality of life as a global measure and to each quality of life domain (physical, psychological, social, and environmental). We expected to see variations in the correlations as some poor health behaviors may initially be perceived to increase short-term quality of life, while simultaneously being associated with poor long-term effects, e.g., substance use [85, 86]. Other health behaviors were expected to show stronger associations with quality of life e.g., physical activity [87], diet [32] sleep [88], meaningful activities [89], social relations [59], sexual health [70]. Analyses were done separately for individuals with neurodevelopmental conditions (self-reported ADHD: 12%, autism:10%, and intellectual disability: 1% of the total sample) and neurotypicals (no self-reported mental or physical health problems) controlling for age (as this differed between individuals with neurodevelopmental conditions (*m* = 37.69, *sd* = 11.69) and neurotypicals (*m* = 46.69, *sd* = 11.68); *t*(1548) = 13.35, *p* < .001).

Third, to examine the relationships among the different health behaviors measured by Lev-s at T1, we conducted a series of correlation analyses using Pearson’s correlation coefficient. Analyses were done separately for individuals with neurodevelopmental conditions and neurotypicals controlling for age. We excepted to find positive associations as healthier behaviors in one domain are generally associated with healthier behaviors in other domains [90]. Benchmarks to define small (*r* = 0.1), medium (*r* = 0.3), and large (*r* = 0.5) effects were used for correlations [91].

Fourth, analyses of covariance (ANCOVA) were used to investigate group differences for each health behavior between individuals with neurodevelopmental conditions controlling for age. Benchmarks to define small (*η*_*p*_^*2*^ = 0.01), medium (*η*_*p*_^*2*^ = 0.06), and large (*η*_*p*_^*2*^ = 0.14) effects were used for ANCOVAs [91].

## Results

First, as can be seen in Table 4, estimates of test-retest reliability (ICC:s) were in the range of .77-.98 for 8 of the ten different health behaviors indicating a good to excellent reliability. ICC for meaningful activities was .74 and .71 for screen health indicating moderate reliability. When investigating ICC on an item level for the three items in meaningful activities, we found a particular low ICC (.52) for the item *Has anyone else commented on how much time you spend on your leisure activities*?, indicating low test-retest reliability. For screen health, it was not possible to investigate ICC as this health behavior only includes one item: *Do you identify with the following statements*? *Others think that you spend too much time on screens*. *It’s difficult to set limits and to adhere to the ones you’ve set*. *You use screens to escape reality/avoid negative feelings*. Furthermore, detailed analyses for each health behavior, including Bland-Altman plots illustrating the measurement consistency and frequency distributions, are provided in the supplementary materials (S2 File). These plots show that the majority of data points fall within the limits of agreement for each behavior, with mean differences close to zero, indicating minimal systematic bias and supporting the reliability of Lev-s across subscales.

**Table 4:** Test-retest analysis for Lev-s.

Health behaviors in Lev-s	Mean (sd) T1	Mean (sd) T2	ICC (95% CI)	SEM
Physical activity	1.75 (.66)	1.73 (.66)	.89 (.85-.92) ***	.22
Diet	1.65 (.71)	1.58 (.73)	.77 (.70-.83) ***	.34
Alcohol	1.64 (.47)	1.66 (.45)	.87 (.83-.90) ***	.17
Tobacco	2.68 (.91)	2.67 (.91)	.98 (.98-.99) ***	.13
Illegal Drugs	1.94 (.26)	1.95 (.29)	.92 (.89-.94) ***	.07
Sleep	1.88 (.68)	1.89 (.71)	.86 (.82-.90) ***	.27
Social relations	2.38 (.61)	2.36 (.60)	.87 (.82-.90) ***	.22
Meaningful activities	2.08 (.53)	2.08 (.59)	.74 (.66-.80) ***	.27
Sexual health	1.05 (1.10)	1.17 (1.17)	.81 (.74-.86) ***	.48
Screen health	1.83 (.83)	1.88 (.82)	.71 (.62-.78) ***	.45

Means, standard deviations (T1-T2), intra-class correlation coefficients with 95% confidence intervals and standard error of measurement for all health behaviors included in Lev-s.

*** *p* <0.001

Second, as shown in Table 5, similar correlations between health behaviors and global quality of life ratings were observed for both individuals with neurodevelopmental conditions and neurotypicals. Generally, healthy behaviors correlated positively with higher global quality of life ratings, except for alcohol, tobacco, and illegal drug use where correlations were small or non-significant. Notably, the patterns of correlations between health behaviors and specific quality of life subdomains varied between the two groups. For neurotypicals, the strongest correlations were in general found in the physical subdomain. In contrast, individuals with neurodevelopmental conditions displayed correlations to a broader range of subdomains of quality of life.

**Table 5:** Partial correlations of Lev-s to quality of life.

Health behaviors in Lev-s	Correlation to global quality of life in WHOQOL-BREF (Pearsons r)		Correlations to quality of life domains (Pearsons r)	
	Neuro-developmental conditions (n = 399)	Neurotypicals (n = 1135)	Neuro-developmental conditions (n = 399)	Neurotypicals (n = 1135)
Physical activity	.21***	.26***	.24*** (physical)	.50***(physical)
Diet	.29***	.35***	.32***(psychological)	.51***(physical)
Alcohol	-.06 ns	.10***	-.12***(environmental)	.50***(physical)
Tobacco	.00 ns	.04 ns	.07 ns	.21***(physical)
Illegal Drugs	-.03 ns	.10***	-.07 ns	.54***(physical)
Sleep	.50***	.47***	.55***(physical)	.73***(physical)
Social relations	.56***	.54***	.57***(social)	.59***(physical)
Meaningful activities	.31***	.46***	.31***(psychological)	.64***(physical)
Sexual health	.29***	.39***	.27***(social)^a^	.42***(social)^a^
Screen health	.21***	.33***	.22***(psychological)	.45***(physical)

Partial correlations, controlling for age, at T1 of each health behavior included in Lev-s to global quality of life and quality of life domains, physical, psychological, social, and environmental, included in WHOQOL-BREF. Partial correlations to both global quality of life and quality of life domains are reported separately for individuals with neurodevelopmental conditions and neurotypicals.

*** *p* <0.001, ns = non-significant.

^a^ Removing the item “How satisfied are you with your sex life?” from the quality of life social subdomain decreased the strength of the correlation to sexual health (*r* = .21, *p* < 0.001) making “physical” the strongest correlated subdomain (*r* = .35, *p* < 0.001) for neurotypicals. For individuals with neurodevelopmental conditions, removing this item decreased the strength of the correlation to sexual health (*r* = .14, *p* < 0.001) making environment the strongest correlated subdomain (*r* = .24, *p* < 0.001)

Third, the matrix with partial correlations presented in Table 6 shows the relationships between all health behaviors assessed in Lev-s reported separately for individuals with neurodevelopmental conditions and neurotypicals. Correlations were found between almost all health behaviors (small to large effect sizes) and were stronger in the neurotypical group. Standing out was the strong associations between illegal drugs and alcohol (*r*s *=* .78-.85, *p* < .001).

**Table 6:** Partial correlation between health behaviors in Lev-s.

	1	2	3	4	5	6	7	8	9
1. Physical activity									
2. Diet	.33***/.48***								
3. Alcohol	.29***/.54***	.30***/.48***							
4. Tobacco	.02ns/.27***	.23***/.26***	.35***/.47***						
5. Illegal Drugs	.19***/.55***	.29***/.50***	.78***/.85***	.34***/.44***					
6. Sleep	.29***/.49***	.37***/.52***	.41***/.56***	.19***/.26***	.35***/.59***				
7. Social relations	.24***/.48***	.32***/.49***	.33***/.57***	.12*/.25***	.29***/.61***	.40***/.56***			
8. Meaningful activities	.41***/.55***	.37***/.51***	.51***/.58***	.19***/.26***	.44***/.59***	.45***/.59***	.45***/.60***		
9. Sexual health	-.02ns/.21***	.15***/.24***	.16***/.25***	.12*/.16***	.19***/.25***	.27***/.31***	.21***/.37***	.19***/.28***	
10. Screen health	.35***/.39***	.28**/.41***	.34***/.40***	.08ns/.13***	.32***/.44***	.32***/.47***	.24***/.42***	.50***/.54***	.14**/.22***

Partial correlation, controlling for age, for the 10 health behaviors in Lev-s at time point 1 reported separately for individuals with neurodevelopmental conditions (n = 399)/ Neurotypicals (n = 1135).

* *p* <0.05

** *p* <0.01

*** *p* <0.001, ns = non-significant

Fourth, small-sized group differences, indicating higher risk for poor health in individuals with neurodevelopmental conditions, were observed for diet, sleep, social relations, and sexual health (see Table 7). Group differences were non-significant for physical activity, meaningful activities or screen health, *F*s < 2.35. Significant, small to large effect-sized group differences (*η*_*p*_^*2*^ = .01-.13), indicating higher risk for neurotypical individuals, were found for all three substance-use behaviors.

**Table 7:** Group comparisons between individuals with neurodevelopmental conditions and neurotypicals.

Health behaviors	Neurodevelopmental conditions (n = 408) Mean (Standard deviation)	Neurotypicals (n = 1142) Mean (Standard deviation)	*F (η*_*p*_^*2*^ *)*
Physical activity	1.22 (.70)	1.28 (.63)	2.35 ns (.002)
Diet	0.90 (.72)	1.12 (.67)	25.38*** (.016)
Alcohol	2.11 (.97)	1.27 (.93)	225.99*** (.128)
Tobacco	2.19 (1.10)	2.02 (.82)	9.19** (.006)
Illegal Drugs	2.28 (.97)	1.60 (.93)	142.80*** (.085)
Sleep	1.11 (.69)	1.41(.64)	59.34*** (.037)
Social relations	1.67 (.76)	1.84 (.61)	17.81*** (.011)
Meaningful activities	1.49 (.68)	1.50 (.64)	.02 ns (.000)
Sexual health	.56 (.93)	.81 (.89)	21.43*** (.014)
Screen health	1.27 (.95)	1.36 (.90)	2.47 ns (.002)

Group comparisons between individuals with neurodevelopmental conditions (ADHD, autism, intellectual disability and neurotypicals. The colors indicate risk level according to Lev-s: “red” high risk for ill health; “yellow” risk for ill health; green healthy behavior.

*** *p* <0.001

** *p* <0.01, ns = non-significant

## Discussion

Health behaviors are significant contributors to physical, social and mental health. Because non-communicable diseases stands for the majority of all deaths globally, there is a great necessity for a fundamental change towards a larger acknowledgement of health behaviors within multiple sectors of health care [31, 32]. In response, we have developed Lev-s, a multifaceted health behavior screening tool in co-operation with several researchers and health care professionals and assessed some of its psychometric properties. Our evaluation demonstrates good to excellent test-retest reliability and satisfactory validity, endorsing Lev-s as a promising instrument for health behavior screening.

### Development of Lev-s

Acknowledging health behaviors is crucial for preventing mental and physical health issues, reducing premature deaths, and easing healthcare system loads. While current instruments evaluate health behavior, they fall short in offering a holistic assessment across the multifaceted domains it encompasses. We crafted the Lev-s by selecting ten health behaviors and creating 33 items striving for a universal applicability to accommodate individuals with a broad range of cognitive and physical abilities. We did this by reviewing the litterateur and consulting experts and healthcare workers in an iterative process. The scoring levels—labelled as green (healthy), yellow (at risk), and red (high risk)—are derived from a comprehensive analysis of contemporary research delineating the correlation between specific health behaviors and health outcomes. Furthermore, we reviewed the literature to make sure that the items and scoring levels were relevant recognizing variation in cognitive and physical abilities, biological sex, gender identity and age.

In this article, we included a special attention to neurodevelopmental conditions, but the development of Lev-s considered other variations in functioning as well. In this context we want to stress the importance to recognize that communication and behavioral change strategies regarding how to address poor health behaviors, are complex and should be tailored to the individual [92]. For instance, a sedentary person with low motivation might benefit more from personalized goals below WHO’s recommendations for physical activity [31] than immediately attempting to meet recommended levels. However, we decided that the purpose of Lev-s is to detect universally poor health behaviors, leaving it up to the healthcare worker and patient to arrive at how to set goals and strategies. It would be unethical and against the Convention on the Rights of Persons with Disabilities, not to convey information and services on an equal basis with others [93]. However, in this context, we reflected on if screening with Lev-s could lead to being discouraged if scoring low across many health behaviors. This might be extra important in the context of disability, considering the various barriers individuals with disabilities face in accessing healthcare and pursuing a healthy lifestyle [94]. These barriers can exacerbate feelings of discouragement and helplessness, especially if additional challenges in the form of poor health behaviors, are revealed through new screening tools. To address this concern, we included pre-use instructions with Lev-s to proactively manage expectations and mitigate potential disappointment (e.g., highlighting health behaviors as malleable and that change of a poor health behavior could lead to well-being). Furthermore, we encourage that the screening with Lev-s is followed by providing adequate support, clear communication about the benefits of the screening, and ensuring that any identified poor health behaviors are addressed with actionable, supportive interventions can help to reduce potential discouragement.

In summary, consistent with the first aim of this study, we have systematically detailed the development of Lev-s. We hope that this description will offer transparency regarding the selection of included health behaviors and the aspects they encompass.

### Psychometric properties

When investigating test-retest reliability of the Lev-s we found good to excellent reliability for most health behaviors. This indicates that the Lev-s consistently measures health behaviors over a short time, supporting its utility for screening purposes in clinical settings. The high test-retest reliability indicates that any observed changes in health behaviors will be due to actual changes rather than measurement inconsistencies. However, future studies may estimate the magnitude of minimal important change to support the clinician in deciding if changes across time are beyond measurement error [95]. While most health behaviors showed good to excellent test-retest reliability, the ICC was slightly lower for Meaningful activities (.74) and screen health (.71). For the health behaviors “Meaningful activities” The item *Has anyone else commented on how much time you spend on your leisure activities*? got the lowest ICC value. This item was inspired by similar questions (e.g., "Has a relative or friend, or a doctor or other health worker been concerned about your drinking or suggested you cut down?"), commonly used in alcohol use assessments to identify problematic behaviors [96]. For individuals with neurodevelopmental conditions such as autism, engaging in specific activities for prolonged periods is common and may attract comments by close relations [97]. As our objective was to develop a screening tool that encompasses an inclusive range of cognitive functioning, we believe it is essential to retain this item. Future studies should consider a more diverse sample that includes a higher proportion of individuals with neurodevelopmental conditions to better capture the intended construct of the item. For the health behavior "Screen health", we opted to ask how many of the four statements about screen behavior the participant agreed with rather than including four separate questions. This decision aimed to limit the number of questions in Lev-s. However, given the moderate ICC value observed, future studies should explore whether formulating the statements as separate questions would enhance test-retest reliability. The current design might place an excessive load on working memory, requiring participants to retain multiple statements simultaneously while considering their applicability, which could impact the consistency of their responses.

While investigations of reliability typically include assessment of internal consistency, this was not calculated for the health behaviors in Lev-s as items do not always aim to measures the same underlying factor/aspect. However, as another indication of reliability, during the development process in a separate study, inter-rater reliability was assessed and demonstrated to be excellent across all health behaviors when final-year students from various healthcare professions filled in Lev-s viewing fictive patient interviews [81]. Overall, the iterative development phase, which involved selecting items and rephrasing them for increased clarity and comprehension, resulted in acceptable levels of reliability.

In this study we have begun investigating validity for Lev-s instrument. We did this by examining the correlations with quality of life, intercorrelations among the different health behaviors in Lev-s and by comparative analyses between individuals with neurodevelopmental conditions (self-reported ADHD, autism and intellectual disability) and neurotypical individuals—which comprises individuals without self-reported psychiatric diagnoses or possibly impairing diseases. We found that seven of the ten health behaviors exhibited a significant correlation with a global assessment of quality of life. Notably, many health behaviors that generally receive less priority in guidelines [98], displayed substantial correlations with global quality of life. In line with previous research, these include sleep [88], social relationships [59], engagement in meaningful activities [89], and sexual health [70], with medium to large-sized correlations both in individuals with neurodevelopmental conditions and neurotypicals. Of the health behaviors that typically receive more attention, positive associations were also found in line with previous research to physical activity [87] and diet [32]. Thus, while demonstrating the anticipated correlations with quality of life, our investigation also shows that utilizing the Lev-s can effectively identify several health behaviors that may significantly improve quality of life. Furthermore, health behaviors can be subject for preventive interventions or by amplifying other existing interventions. Recognizing multiple health behaviors may increase the likelihood of identifying health behaviors relevant to the individual. This consideration is crucial, given that changing health behaviors has proven to be a challenge [99] and finding areas for which the patient’s intrinsic motivation is high enough for active participation could be important for increasing participation in their health situation (Michaelsen & Esch, 2023). Interestingly, our findings indicate that substance use, including alcohol, tobacco, and illegal drugs, does not correlate or display small-sized correlations with lower global quality of life for both groups. While substance use disorders are ultimately linked to a diminished quality of life [100] recreational use can be perceived as rewarding short-term [86] which may account for the positive perception observed among many individuals. However, when investigating the strongest correlating quality of life subdomain in WHOQOL-BREF to Lev-s, the non-significant or weak associations to substance use using global quality of life, become medium or large-sized for neurotypicals. Notably, the patterns of correlations between health behaviors and specific quality of life subdomains varied between the two groups and were stronger for neurotypicals. The high correlations with the physical domain are supported by the inclusion of items that cover both physical abilities and sleep, which are among the most important factors for well-being [101]. We speculate that individuals with neurodevelopmental conditions may experience a more complex interplay of biological, psychological, and social factors, which could affect how health behaviors influence their quality of life [102]. These factors might include varying levels of social support, different coping mechanisms, and unique psychological profiles that could weaken or obscure the direct impact of health behaviors on quality of life. It is important to note that the neurotypical participants in this study were highly functioning, reporting no mental or physical health problems. This characteristic might have amplified the observed differences. Further research is needed to explore these differences in greater detail.

Overall, the pattern of intercorrelations suggests that healthier behaviors in one domain are generally associated with healthier behaviors in other domains, supporting the holistic nature of health behavior engagement [90]. The strong association between illegal drug use and alcohol consumption (*r*s = .78-.85, p < .001) is in accordance with previous research indicating high co-occurrence between these behaviors [103]. Notably, the stronger correlations found in the neurotypical group may indicate that these individuals exhibit more consistent and predictable patterns of health behaviors [101, 104]. As with associations to quality of life, individuals with neurodevelopmental conditions may experience a more complex interplay of biological, psychological, and social factors, which can affect how health behaviors are associated with each other (Bolte et al., 2021). This complexity might reduce the strength of inter-correlations of health behaviors due to the varying influences and interactions of these multifaceted factors.

Moreover, we assessed health behavior levels in individuals with neurodevelopmental conditions in comparison to neurotypical counterparts. This comparative analysis was undertaken despite Lev-s primarily being designed for screening purposes rather than for detecting differences. Still, we expected results of the screening to be roughly in line with prior research. On average, the neurotypical individuals fall within the yellow level, signifying a “risk status” according to Lev-s. This confirms the general view of that addressing health behaviors could be important for current and future health for the vast majority [1, 2], including neurotypicals which in this study comprises individuals with no self-reported mental or physical health problems. Both individuals with neurodevelopmental conditions and neurotypicals were found in the green, “healthy habits field” for tobacco use (e.g., does not use tobacco or quit more than six months ago). However, this is consistent with that Sweden exhibits one of the lowest levels of smoking in Europe [105]. It should be noted that tobacco use in Lev-s includes consumption of cigarettes, pipes, cigars, cigarillos, hookahs, as well as smokeless tobacco, snus and chewing tobacco. E-cigarettes and other non-tobacco products, are not included in this definition. While smoking tobacco is on decline, alternative forms of nicotine use without tobacco is on the rise in Sweden [106]. Consistent with prior research [71, 107] the mean score for sexual health for both individuals with neurodevelopmental conditions and neurotypicals was low, indicating a “high-risk” status. Importantly, the Lev-s encompasses not just satisfaction but also safety and functional impairments. Instead of averaging these three aspects, we chose to use the minimum score to represent this health behavior, as addressing any of these aspects could significantly improve sexual health. Acknowledging safety and functional impairment is in line with suggestions from a large-scale study showing that close to half of the female population has experienced sexual harassments and more than a third sexual abuse. The same study also confirm the importance of including functional impairments [108]. Including not only satisfaction but also safety and functional impairment, could be a partial explanation to why this area is red i.e., “high risk for ill-health”/ high gain if changed.

In line with previous research, group differences, indicating higher risk for poor health behaviors in individuals with neurodevelopmental conditions, were observed for diet [37–39], sleep [55], social relations [62], and sexual health [72]. We found the similar group levels for health behaviors meaningful activities and screen health difficult to set in context as previous research suggest the need for more homogenous definitions and taking publication bias into account [68, 78]. However, based on reports from health care professionals participating in the development of Lev-s, individuals with neurodevelopmental conditions exhibit both high and low engagement in these health behaviors, and we therefore expected large variability.

While variability was expected to be large for physical activity levels as well, the lack of differences between individuals with neurodevelopmental conditions and neurotypicals was unexpected based on previous studies [27–29]. This unexpected result might be due to specific characteristics of our study population, scoring levels or the choice of measurement items. In our study, individuals with neurodevelopmental disorders did not differ significantly from neurotypicals in socio-economic variables. The lack of group differences aligns with findings from another Swedish study, which reported that differences between individuals with neurodevelopmental disorders and neurotypicals diminished when controlling for socio-economic factors [109]. Importantly, Lev-s did not fail to detect low physical activity levels in individuals with neurodevelopmental conditions. Instead, it found that neurotypicals also exhibited similarly low levels of physical activity. It is possible that a more fine-grained scoring could have resulted in detectable group differences. However, the primary purpose of Lev-s is to provide a first screening and identify unhealthy behaviors. Thus, not detecting unhealthy physical activity (i.e., false negative) would have been more problematic. The items for physical activity used in this study were based on recommendations from the World Health Organization [31] encompassing vigorous intensity, moderate intensity, and sedentary behavior. At this point, we find no support for changing the included items in Lev-s. However, the limited number of studies investigating physical activity levels in adults with neurodevelopmental disorders underscores the need for further research in this area to better elucidate the findings of this study.

Finally, significant differences, but where the neurotypical population showed unhealthier behaviors than the neurodevelopmental group, were found for the substance use (tobacco, alcohol, illegal drugs). Previous research has shown that substance use may be more pronounced in individuals with neurodevelopmental conditions than in neurotypical individuals [44, 45, 52]. As items used in Lev-s are very similar to well established assessments for substance use, we lean towards that this finding, as with physical activity, might be explained by that socio-economic factors between the two groups did not differ [110]. We expect that we would have found higher levels of substance use in a sample of including neurodevelopmental conditions when recruited from a strictly clinical context as symptom severity and functional impairments tend to be higher in these settings [111]. In sum, overall, the Lev-s show satisfactory validity in the form of expected results for associations to quality of life, intercorrelations between health behaviors and group comparisons.

### Limitations and future research

Interpreting the promising findings in this first psychometric evaluation of Lev-s requires acknowledging certain limitations. First, the sample characteristics likely influenced the results. The demographic primarily consisted of women with high socio-economic status, as indicated by high levels of education and employment. This may have introduced a bias, potentially overstating the stability of the Lev-s in the test-retest analysis. Additionally, as mentioned in the discussion, the non-clinically referred population in this study might have affected the results. Therefore, it is crucial to include a broader range of specific populations in the continued evaluation of Lev-s to validate the tool’s psychometric properties comprehensively. This is essential for making Lev-s applicable in various healthcare settings across individuals with diverse cognitive and physical functioning.

Second, more dimensions of validity could further substantiate Lev-s. Despite reviewing scientific literature and consulting experts to determine the aspects to include, comparing Lev-s with established screening instruments tailored to specific health behaviors would enhance its psychometric value. However, including an excessive number of questionnaires in this study is challenging, mirroring the very issue we aim to resolve by developing a concise, global screening tool for health behaviors. Moreover, conducting a factor analysis typically provides theoretical justification, but for several health behaviors, we included only one item per aspect (e.g., sexual health included one item each for satisfaction, impairment, and safety), limiting the assessment of the internal structure. While experts have reviewed the included aspects and items, future studies could conduct participant interviews to ensure the items effectively capture the intended aspects. Including participant perspectives from different cognitive and physical disabilities could also help identify important aspects to include for the different health behaviors in future revisions.

Third, as Lev-s encompasses no less than ten health behaviors, new research findings and guidelines necessitate continuously revising what aspects and items to include. Revisiting and potentially revising Lev-s is essential to ensure it remains current and scientifically sound. Fourth, the scoring levels of the three-color fields—red, yellow, and green—symbolize high risk for ill health/high gain if changed, risk for ill health, and healthy habits. These were set based on what is suggested by the scientific literature and guidelines. However, including a longitudinal design where unhealthy behaviors could be linked to ill health would substantiate these levels further. While some health behaviors and the cut-offs between the three-color fields rest on a wealth of research, other areas such as screen health would benefit from further validation. Using a longitudinal design could also enable investigating how acknowledging health behaviors with Lev-s affects patients and healthcare staff. This final limitation will be important for revising pre-use instructions to mitigate possible distress.

## Conclusion

In response to the urgent requirement for an enhanced emphasis on health behaviors in multiple healthcare sectors, we have developed and evaluated the Lev-s instrument. When developing Lev-s, a special focus has been on suitability for wide spectrum of physical, psychological and social functioning. Our evaluation yields evidence of satisfactory reliability and validity, thereby providing preliminary support for the use of Lev-s as an effective screening tool for health behaviors in groups with and without disabilities.

## Supporting information

S1 File (DOCX)

S2 File (PPTX)
