# Supplementary material for: Global screening of health behaviors: Introducing Lev-screening (Lev-s)–development and psychometric evaluation
Source: PLoS One. 2024 Dec 26;19(12):e0315565. doi: 10.1371/journal.pone.0315565 (PMC11670939; doi:10.1371/journal.pone.0315565)
Supplement: S1 File — (DOCX) [file pone.0315565.s001.docx]

**S1**

**Assessment of Global Health Behaviors (Lev-s)**

**Background**

This screening instrument is designed for a preliminary survey of health behaviors and to identify where changes may be needed. It's meant to complement, not replace, more detailed assessments in each area.

**Adjustment of instruction**

You may need to adapt questions to enhance understanding, for example, by explaining more, adjusting language, asking follow-up questions, or referring to previously shared information.

**Scoring**

Choose the best answer option, potentially reading them aloud to facilitate understanding. On the scale used (0 to 3), 3 points indicate the healthiest and 0 points the least healthy. Scores are plotted on an overview figure to show where there's room for healthier behaviors, based on either the average or the minimum. An instruction for how to calculate the score for each area is available in the end of this document. An Excel template is available for assistance.

**Feedback to the Participant**

Show results by marking dots for each health behaviors on the overview figure. It's important to consider that some individuals may be discouraged by their results. Emphasize that health behaviors are not fixed traits but that they can be changed and congratulate them on starting this process. Conduct a follow-up of those disappointed by their outcomes.

**Participant Information**

Changing your health behaviors can significantly transform your life in a positive way. The initial step is to assess your current health behaviors. It's important to remember that health behaviors are not fixed traits; they are the habits you have at the moment, but they can be changed. Even if areas are identified where you could live more healthily, the decision to work on any particular health behaviors is entirely up to you.

The health behaviors questionnaire includes questions that may be sensitive for some individuals. The areas addressed include physical activity, diet, alcohol, tobacco, illegal drugs, sleep, social relations, meaningful activities, sexual health, screen health. Before each section, you'll be asked if it's okay to proceed with the questions, and you have the option to skip sections or individual questions. May I ask about your health behaviors?

**Physical activity**

Is it okay if I ask about your physical activity? All questions are about the past month.

**1. How much time do you typically spend on physical exercise each week? Physical exercise refers to activities that make you breathless and/or increase your heart rate, like running, aerobics, and ball sports.**

Less than 30 minutes (half an hour) 0 points

30-75 minutes (half an hour to a little more than an hour) 1 point

75-150 minutes (1-2.5 hours) 2 points

More than 150 minutes (more than 2.5 hours) 3 points

**2. How much time do you spend on everyday physical activities each week? This includes activities that increase your heart rate, make you breathless or slightly warm, like brisk walking, cycling, or vacuuming.**

Less than 30 minutes (half an hour) 0 points

30-150 minutes (half an hour to 2.5 hours) 1 point

150-300 minutes (2.5-5 hours) 2 points

More than 300 minutes (more than 5 hours) 3 points

**3. What portion of your waking hours do you spend sitting/lying still during a day? On average.** *A person who sleeps 8 hours is awake for 16 hours.*

Almost all day (around 15h) 0 points

75% of the day (around 12h) 1 point

50% of the day (around 8h) 2 points

25% of the day (around 4h) 3 points

**4. How often do you interrupt sitting with movement? For example, cleaning, going to the restroom, walking around for a while.**

Every fourth hour or less often 0 points

Every other hour 1 point

Once an hour 2 points

Once every half hour 3 points

**Diet**

Is it okay if I ask about your eating habits? All questions concern the last month.

**5. How often do you eat vegetables and/or root vegetables (fresh, frozen, or cooked)?**

Once a week or less 0 points

A few times a week 1 point

Once a day 2 points

Twice a day or more 3 points

**6. How often do you eat fruit and/or berries (fresh, frozen, canned, etc.)?**

Once a week or less 0 points

A few times a week 1 point

Once a day 2 points

Twice a day or more 3 points

**7. How often do you eat fish or seafood as a main dish, in a salad, or as a topping? If you are vegan/vegetarian, you can substitute fish and seafood with plant-based alternatives (such as soy-based products, nuts, avocado, beans/lentils).**

A few times a month or less 0 points

Once a week 1 point

Twice a week 2 points

Three times a week or more 3 points

**8. How often do you eat pastries, chocolate/candy, chips, or drink soda/juice?**

Twice a day or more 0 points

Once a day 1 point

A few times a week 2 points

Once a week or less 3 points

**9. How often do you eat breakfast?**

Once a week or less 0 points

A few times a week 1 point

Almost every day 2 points

Daily 3 points

**10. Sometimes we eat or avoid eating for reasons other than hunger. How often do you eat/do not eat because you feel stressed, anxious, bored, sad, or excited?**

It happens very often, every day 0 points

It happens often, a few times a week 1 point

It happens occasionally, a few times a month 2 points

It happens rarely/never 3 points

**Alcohol**

Is it okay if I ask about your alcohol habits? All the questions I will ask are about the last month.

**11. How often do you drink alcohol?**

4 times a week or more 0 points

2-3 times a week 1 point

2-4 times a month 2 points

Once a month or less/never 3 points

**12. How many drinks do you typically have on a day when you drink alcohol?** *Examples of a standard drink include 12-15 cl of wine, 2 x 33 cl of light alcohol beer, 50 cl of medium alcohol beer, 33 cl of strong alcohol beer, 4 cl of spirits.*

7 or more standard drinks 0 points

5-6 standard drinks 1 point

3-4 standard drinks 2 points

1-2 standard drinks or fewer/never drink 3 points

**13. How often do you drink, if you are a woman, 4 standard drinks or more, and if you are a man, 5 standard drinks or more on one occasion?** *Examples of a standard drink include 12-15 cl of wine, 2 x 33 cl of light alcohol beer, 50 cl of medium alcohol beer, 33 cl of strong alcohol beer, 4 cl of spirits.*

Daily or almost daily 0 points

Every week 1 point

Every month 2 points

Never or less often than once a month 3 points

**Tobacco**

Tobacco use includes consumption of cigarettes, pipes, cigars, cigarillos, hookahs, as well as smokeless tobacco, snus and chewing tobacco. E-cigarettes are not included in this definition.

Is it okay if I ask about your tobacco habits?

**14. Do you smoke or use any form of tobacco?**

Yes, currently smoke/use tobacco 0 points

Yes, when partying 1 point

No, but quit less than 6 months ago 2 points

No, never or quit more than 6 months ago 3 points

**15. How much tobacco do you currently use?** *If multiple forms of tobacco are used, the usage is combined.*

Use tobacco several times daily 0 points

Use tobacco, once a day 1 point

Use tobacco, but not daily 2 points

Do not use tobacco 3 points

**Illegal drugs**

By "drugs," we refer to both illegal substances and medicinal drugs used for non-medicinal purposes.

Is it okay if I ask about your drug habits?

**16. Have you ever taken a drug or misused medical substances (excluding alcohol), and if so, when was the last time you used drugs?**

Yes, less than two weeks ago 0 points

Yes, less than three months ago 1 point

Yes, less than one year ago 2 points

No, or more than a year ago 3 points

**17. How often in the past year have you used drugs or misused medical substances?**

2-3 times a week or more 0 points

2-4 times a month 0 points

Once a month or less often 1 point

Never 3 points

**Sleep**

Is it okay if I ask about your sleep? All questions pertain to the last month.

**18. How many hours do you usually sleep per night?** *If you find it hard to know, it may help to write down when you go to bed, fall asleep and wake up.*

Less than 5 hours or more than 10 hours 0 points

5-6 hours or 9-10 hours 1 point

6-7 hours 2 points

7-9 hours 3 points

**19. Do you have problems with any of the following: difficulty falling asleep, waking up during the night, or waking up too early in the morning?** *You can segment the question into three parts: "Do you have difficulty falling asleep?" "Do you have problems with waking up during the night?" "Do you have problems with waking up too early in the morning?" Then, combine how often this happens.*

Yes, 4 or more times a week 0 points

Quite often, 2-3 times a week 1 point

Quite rarely, 2-4 times a month 2 points

No, once a month or less 3 points

**20. Do you go to bed and get up at roughly the same time every day? Consider a difference of more than one hour in bedtime/rising time, including weekends***. By 'the same times,' we mean differences of more than one hour in bedtime or waking time. This also includes any variations between weekdays and weekends.*

No, very irregular sleeping patterns 0 points

Rarely, deviate from sleep routines 3-4 days/week 1 point

Often, deviate from sleep routines 1-2 days/week 2 points

Yes, very regular sleeping patterns 3 points

**21. Do you feel you can manage throughout the whole day?** *"Manage" means being able to carry out the day and planned activities. The opposite could be that you cancel plans or carry them out carelessly because you are tired due to lack of sleep.*

No 0 points

Sometimes, 3-4 days a week 1 point

Often, 5-6 days a week 2 points

Yes 3 points

**22. How satisfied are you with your sleep on a scale from 0-3 where 0 is "Dissatisfied" and 3 is "Satisfied"?**

(0) Dissatisfied (wish for significant change) 0 points

(1) Fairly dissatisfied (desire for change) 1 point

(2) Fairly satisfied (comfortable but open to minor changes) 2 points

(3) Satisfied (no or only marginal changes desired) 3 points

**Social relations**

Is it okay if I ask about your social relationships? All questions are about the last month.

**23. Do you have someone you really trust?**

No 0 points

Don't know, maybe 1 point

Think so 2 points

Yes 3 points

**24. How often do you feel lonely?**

Always, or 4 times a week or more 0 points

Often, 2-3 times a week 1 point

Sometimes, 2-4 times a month 2 points

Never, or less than once a month 3 points

**25. How satisfied are you with your friends/relationships on a scale from 0-3 where 0 means "dissatisfied" and 3 means "satisfied"?** *Dissatisfaction can relate to a wish to have more or closer relationships.*

(0) Dissatisfied (wish for significant change) 0 points

(1) Fairly dissatisfied (desire for change) 1 point

(2) Fairly satisfied (comfortable but open to small changes) 2 points

(3) Satisfied (no or only marginal changes desired) 3 points

**26. How many friends do you have outside of your family? If you have online friends, they count if you also meet physically (IRL). You decide who counts as a friend.**

None 0 points

One 1 point

Two 2 points

Three or more 3 points

**Meaningful activities**

Is it okay if I ask about the activities you like to do? All questions relate to the last month.

**27. How often do you engage in a leisure interest (sports/hobby/interest) that makes you feel good? Feeling good means it makes you happy or calm.**

Does not occur, or less than once a month 0 points

Quite rarely, 2-4 times a month 1 point

Quite often, 2-3 times a week 2 points

Yes, 4 times a week or more 3 points

**28. How satisfied are you with what you do during the day on a scale from 0-3 where 0 means "dissatisfied" and 3 means "satisfied"? You can include work/occupation if it contributes to a sense of fulfillment.**

(0) Dissatisfied (desire for significant change) 0 points

(1) Fairly dissatisfied (some desire for change) 1 point

(2) Fairly satisfied (comfortable but open to small changes) 2 points

(3) Satisfied (no or only marginal changes desired) 3 points

**29. Has anyone else commented on how much time you spend on your leisure activities? For example, screen time.**

Yes, others think I often spend too much time 0 points

Yes, sometimes others think I spend too much time 1 point

Very rarely, but it has happened 2 points

No, (or only positive comments) 3 points

**Sexual health**

Sexuality can be an important part of our well-being and pleasure, involving activities with oneself, another person, or online, and is unique to each individual in terms of desire and preferences.

Is it okay if I ask about your sexual health? The first two questions concern the past year.

**30. How satisfied are you with your sex life on a scale from 0-3, where 0 is "dissatisfied" and 3 is "satisfied"?**

(0) Dissatisfied (desire for significant change) 0 points

(1) Fairly dissatisfied (some desire for change) 1 point

(2) Fairly satisfied (comfortable but open to small changes) 2 points

(3) Satisfied (no or only marginal changes desired) 3 points

**31. Is sex difficult for you: do you experience pain, have a disease, or any other barrier that affects your ability to have sex?**

Yes, my ability to have sex is negatively affected by this 0 points

Yes, but I am satisfied with it nonetheless 3 points

No, my ability to have sex is not hindered by this 3 points

**32. Have you ever experienced sex that was in any way distressing, such as violent, degrading, or non-consensual?**

Yes, and I have not received help or support 0 points

No, but I would like help to feel safer 1 point

Yes, but I have received help and do not need any more 3 points

No 3 points

**Screen health**

Screen time includes activities like gaming, using social media, browsing the internet, and watching TV/movies/series. While screen time can be engaging and enjoyable, it should not negatively impact other health behaviors.

Is it okay if we ask about your screen health?

**33. Do you identify with the following statements?**

- Others think that you spend too much time on screens.
- It's difficult to set limits and to adhere to the ones you've set.
- You feel that your screen time prevents you from doing things you should do (like sleeping, studying, working, engaging in interests, physical activity, meeting friends).
- You use screens to escape reality/avoid negative feelings.

I identify with all the statements 0 points

I identify with several statements 1 point

I identify with one statement 2 points

I do not identify with any of the statements 3 points

**Scoring**

| Physical Activity | Mean (q1 + q2 + q3 + q4) | Score: |
| --- | --- | --- |
| Diet | Lowest value of mean Nutrition/regularity or Eating behavior | Score: |
| *Nutrition/regularity* | Mean (q5 + q6 + q7 + q8 + q9) | *Score:* |
| *Eating Behavior* | (q10) | *Score* |
| Alcohol | Mean (q11 + q12 + q13) | Score: |
| Tobacco | Mean (q14 + q15) | Score: |
| Illegal Drugs | Mean (q16 + q17) | Score: |
| Sleep | Mean (q18 + q19 + q20 + q21 + q22) | Score: |
| Social relations | Mean (q23 + q24 + q25 + q26) | Score: |
| Meaningful activities | Mean (q27 + q28 + q29) | Score: |
| Sexual Health | Lowest value of (q30, q31, or q32) | Score: |
| Screen Health | (q33) | Score: |
