# Supplementary material for: Global screening of health behaviors: Introducing Lev-screening (Lev-s)–development and psychometric evaluation
Source: PLoS One. 2024 Dec 26;19(12):e0315565. doi: 10.1371/journal.pone.0315565 (PMC11670939; doi:10.1371/journal.pone.0315565)
Supplement: S2 File — (PPTX) [file pone.0315565.s002.pptx]

## Slide 1
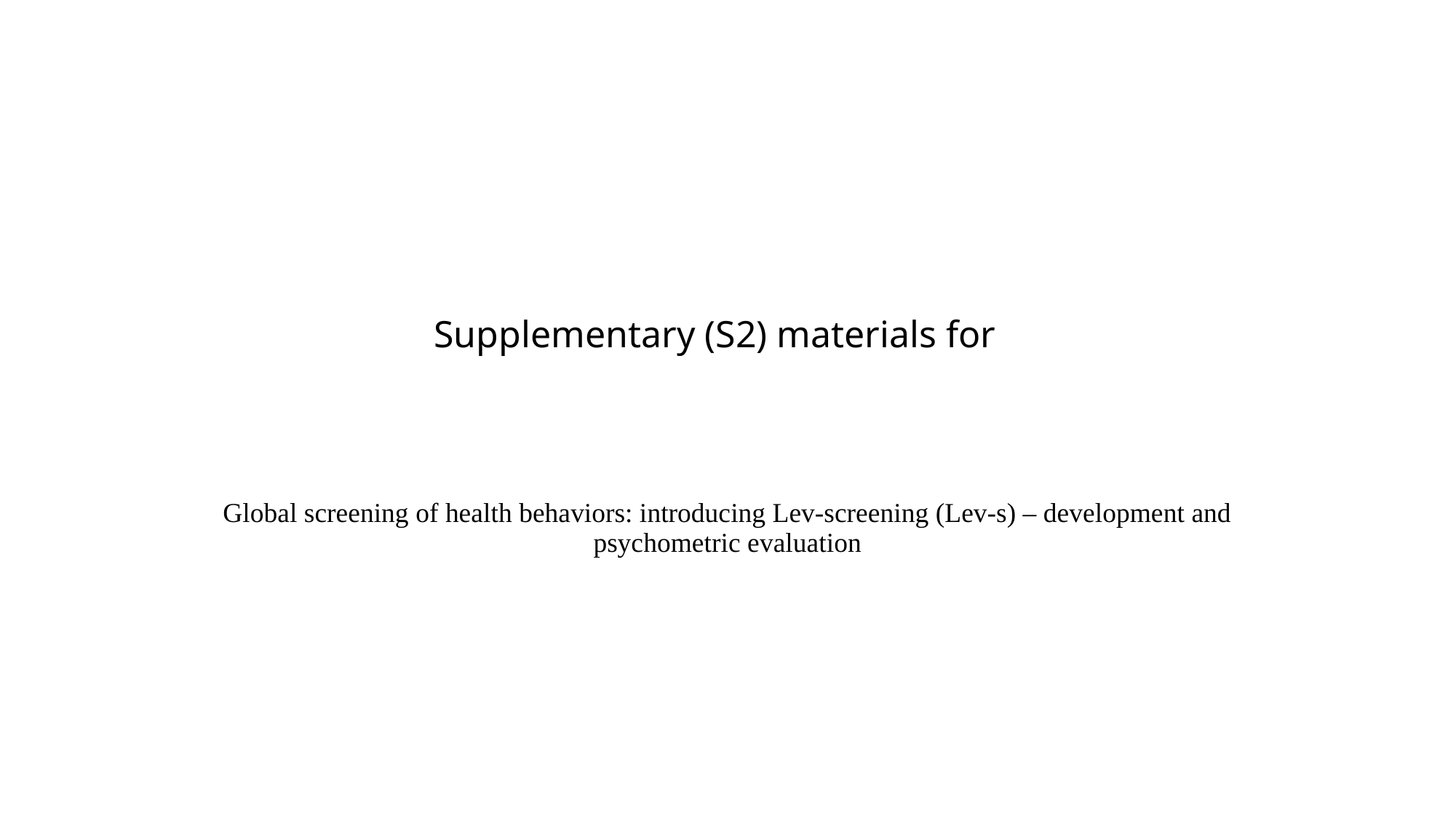

Supplementary (S2) materials for
# Global screening of health behaviors: introducing Lev-screening (Lev-s) – development and psychometric evaluation

## Slide 2
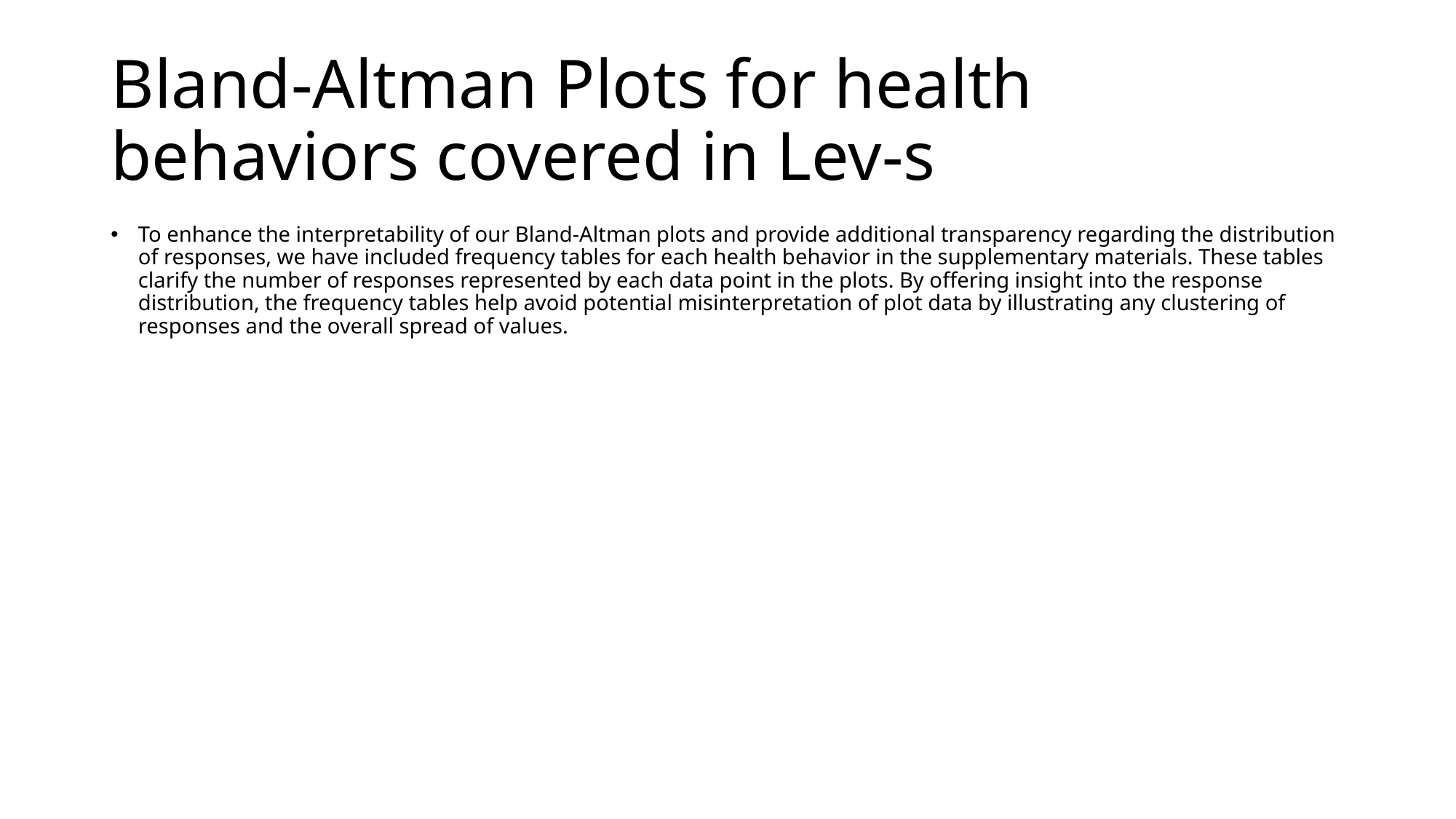

# Bland-Altman Plots for health behaviors covered in Lev-s
To enhance the interpretability of our Bland-Altman plots and provide additional transparency regarding the distribution of responses, we have included frequency tables for each health behavior in the supplementary materials. These tables clarify the number of responses represented by each data point in the plots. By offering insight into the response distribution, the frequency tables help avoid potential misinterpretation of plot data by illustrating any clustering of responses and the overall spread of values.

## Slide 3
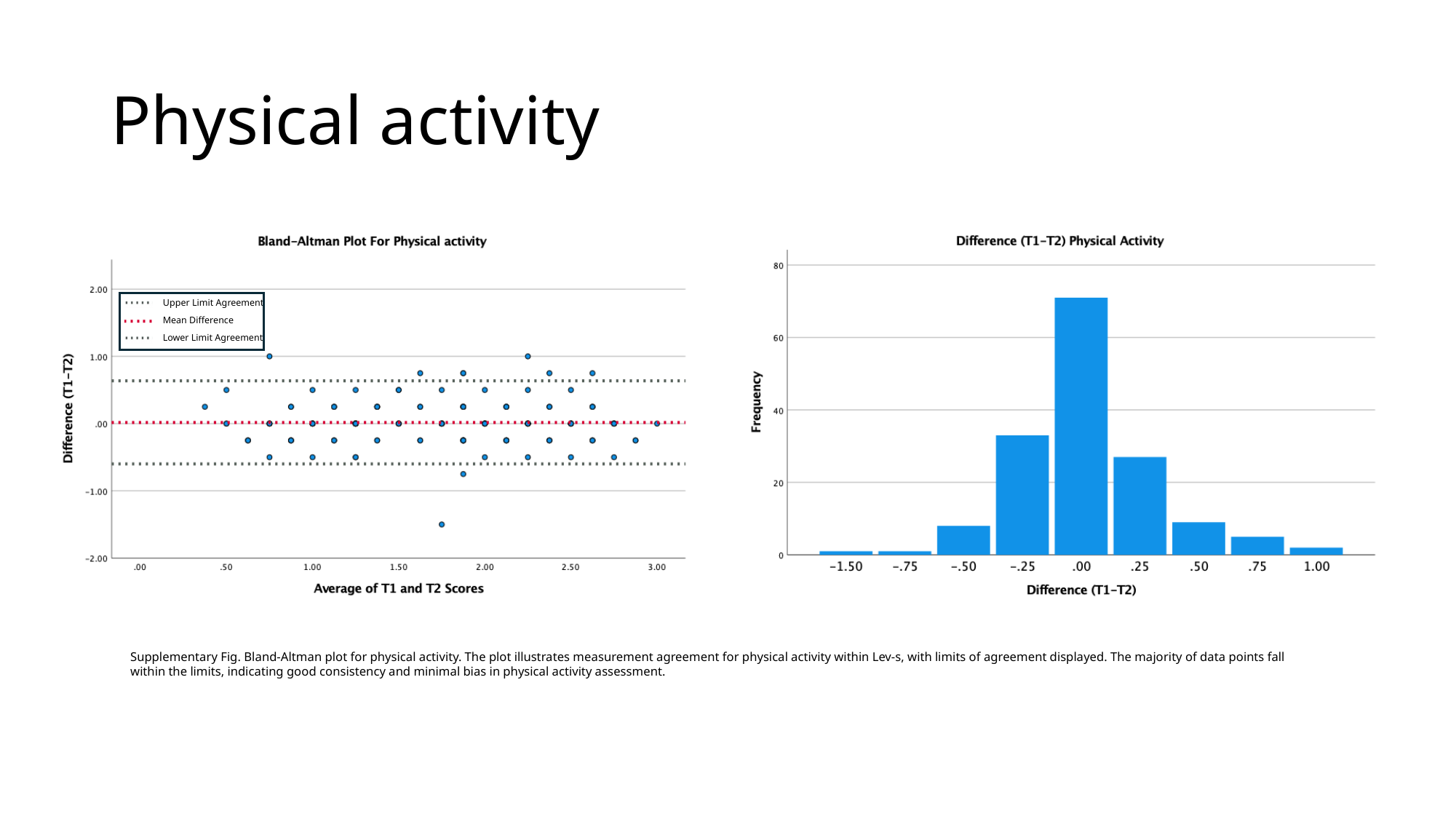

# Physical activity
Upper Limit Agreement
Mean Difference
Lower Limit Agreement
Supplementary Fig. Bland-Altman plot for physical activity. The plot illustrates measurement agreement for physical activity within Lev-s, with limits of agreement displayed. The majority of data points fall within the limits, indicating good consistency and minimal bias in physical activity assessment.

## Slide 4
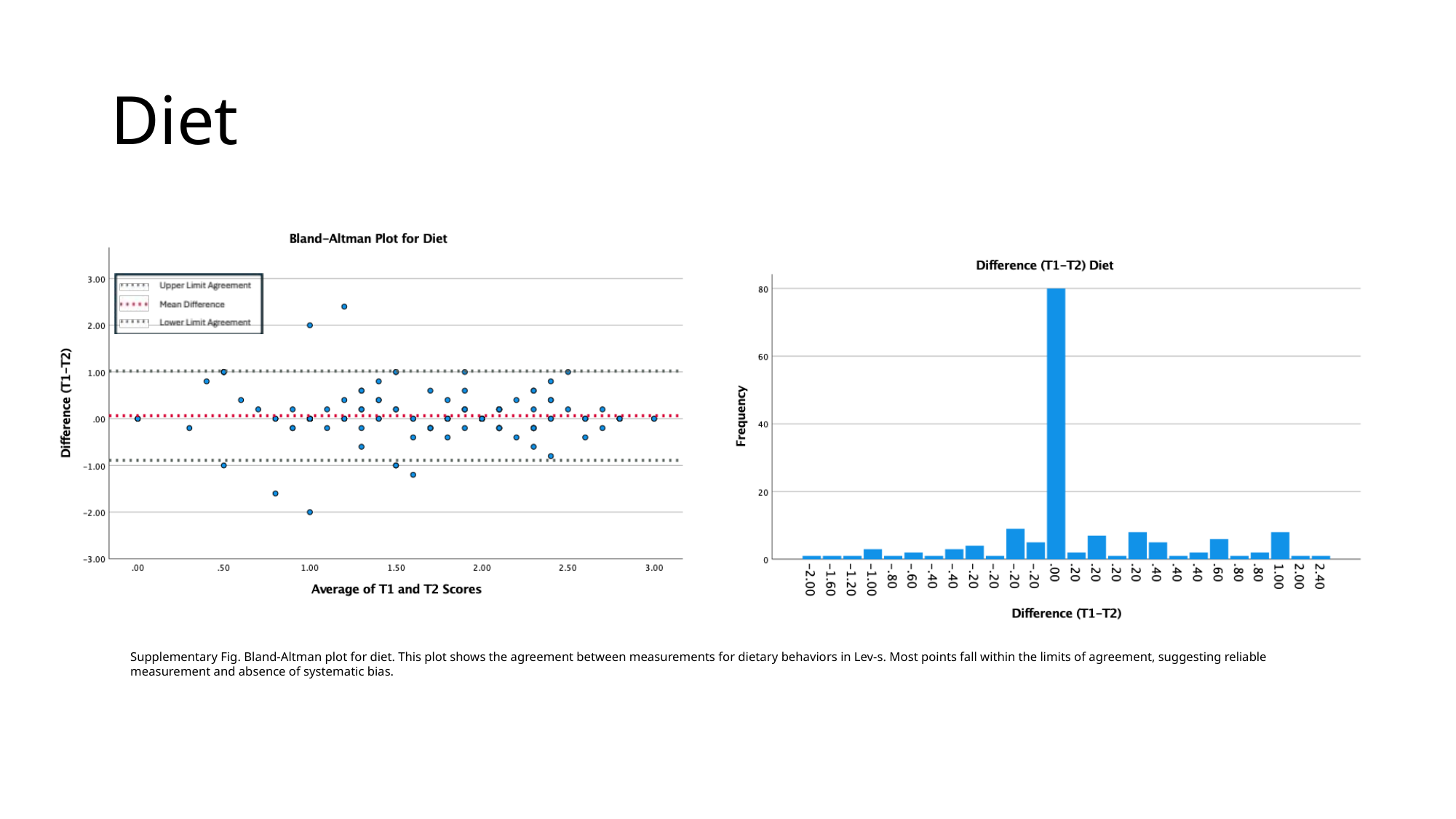

# Diet
Supplementary Fig. Bland-Altman plot for diet. This plot shows the agreement between measurements for dietary behaviors in Lev-s. Most points fall within the limits of agreement, suggesting reliable measurement and absence of systematic bias.

## Slide 5
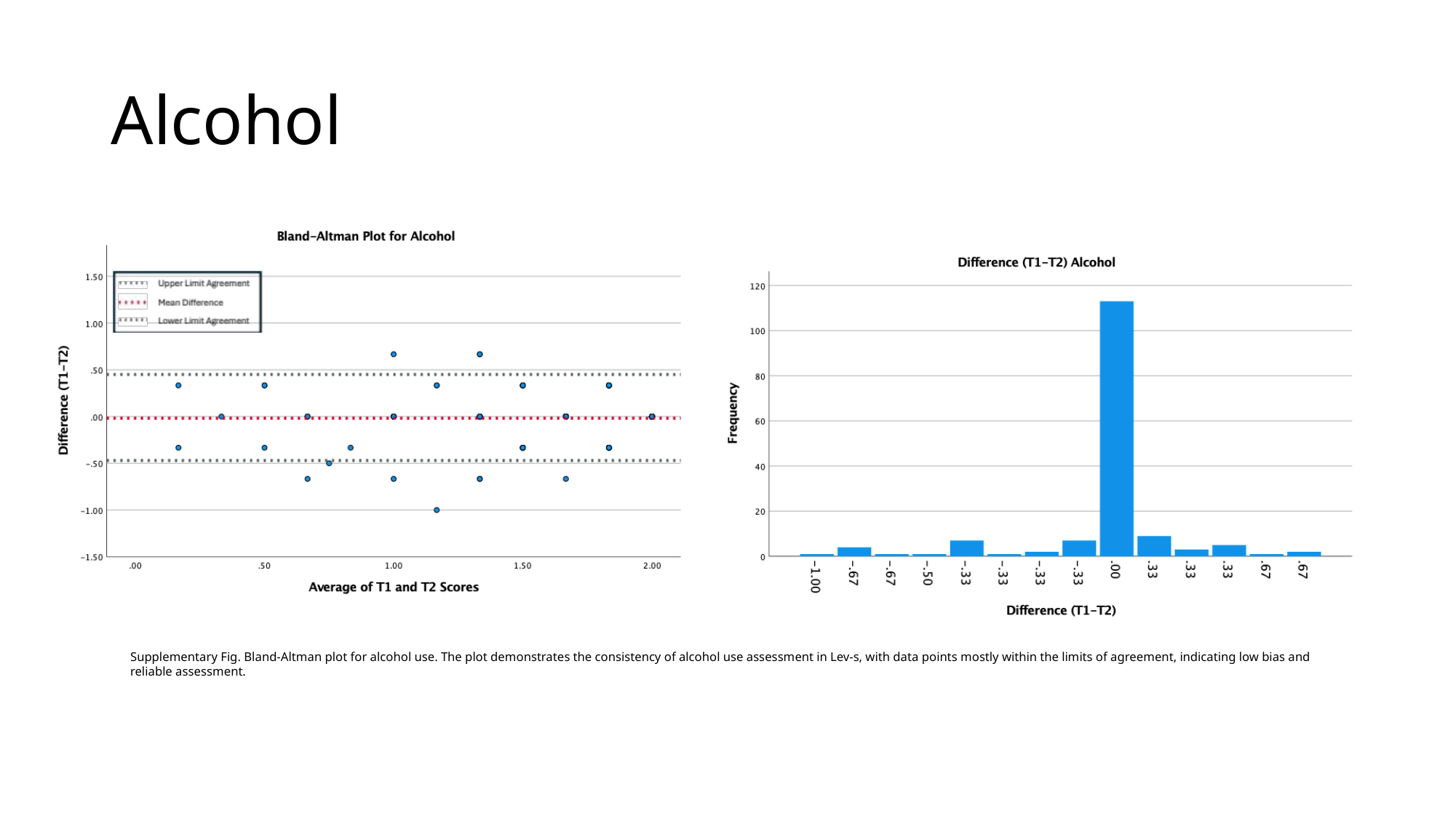

# Alcohol
Supplementary Fig. Bland-Altman plot for alcohol use. The plot demonstrates the consistency of alcohol use assessment in Lev-s, with data points mostly within the limits of agreement, indicating low bias and reliable assessment.

## Slide 6
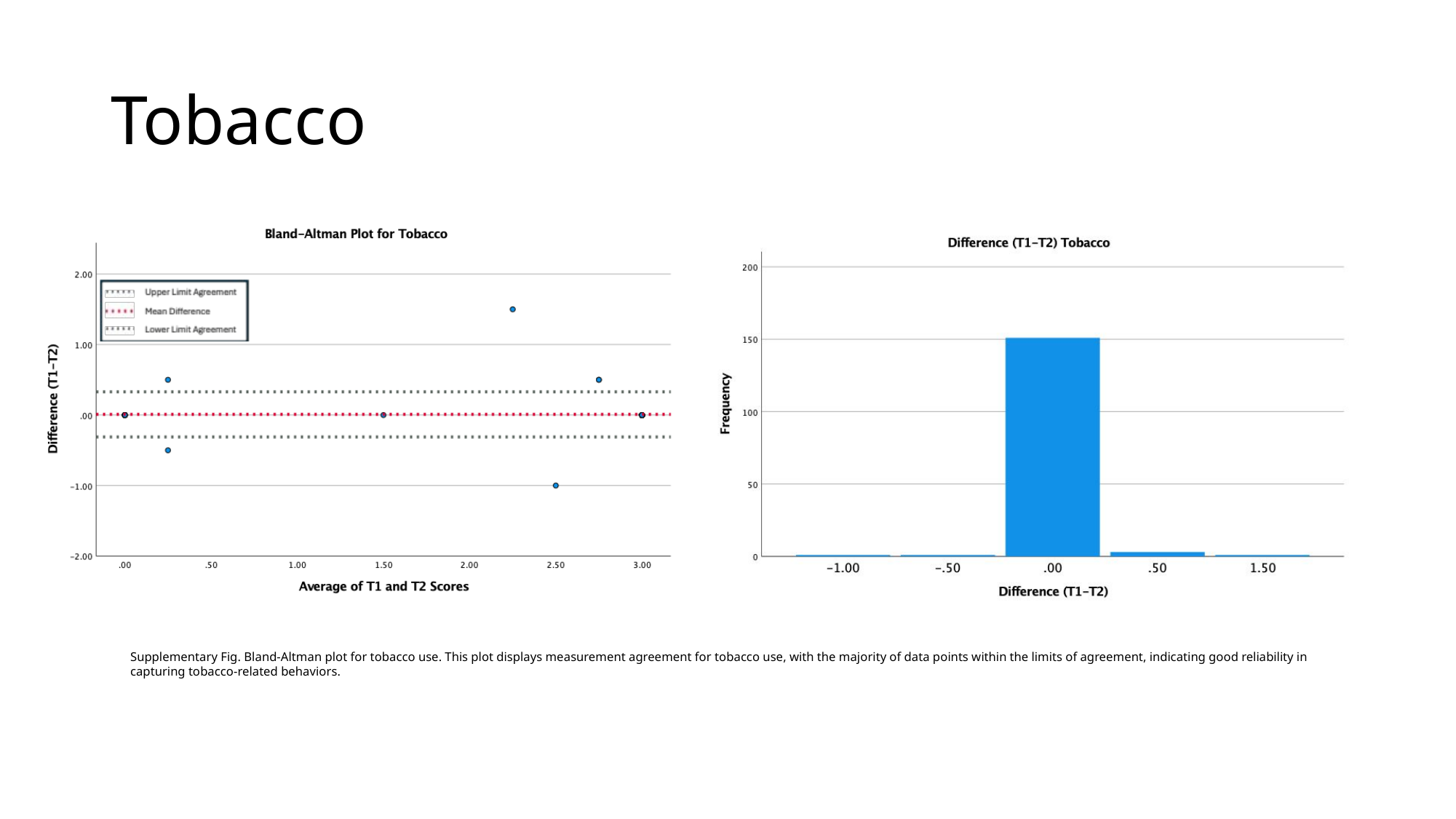

# Tobacco
Supplementary Fig. Bland-Altman plot for tobacco use. This plot displays measurement agreement for tobacco use, with the majority of data points within the limits of agreement, indicating good reliability in capturing tobacco-related behaviors.

## Slide 7
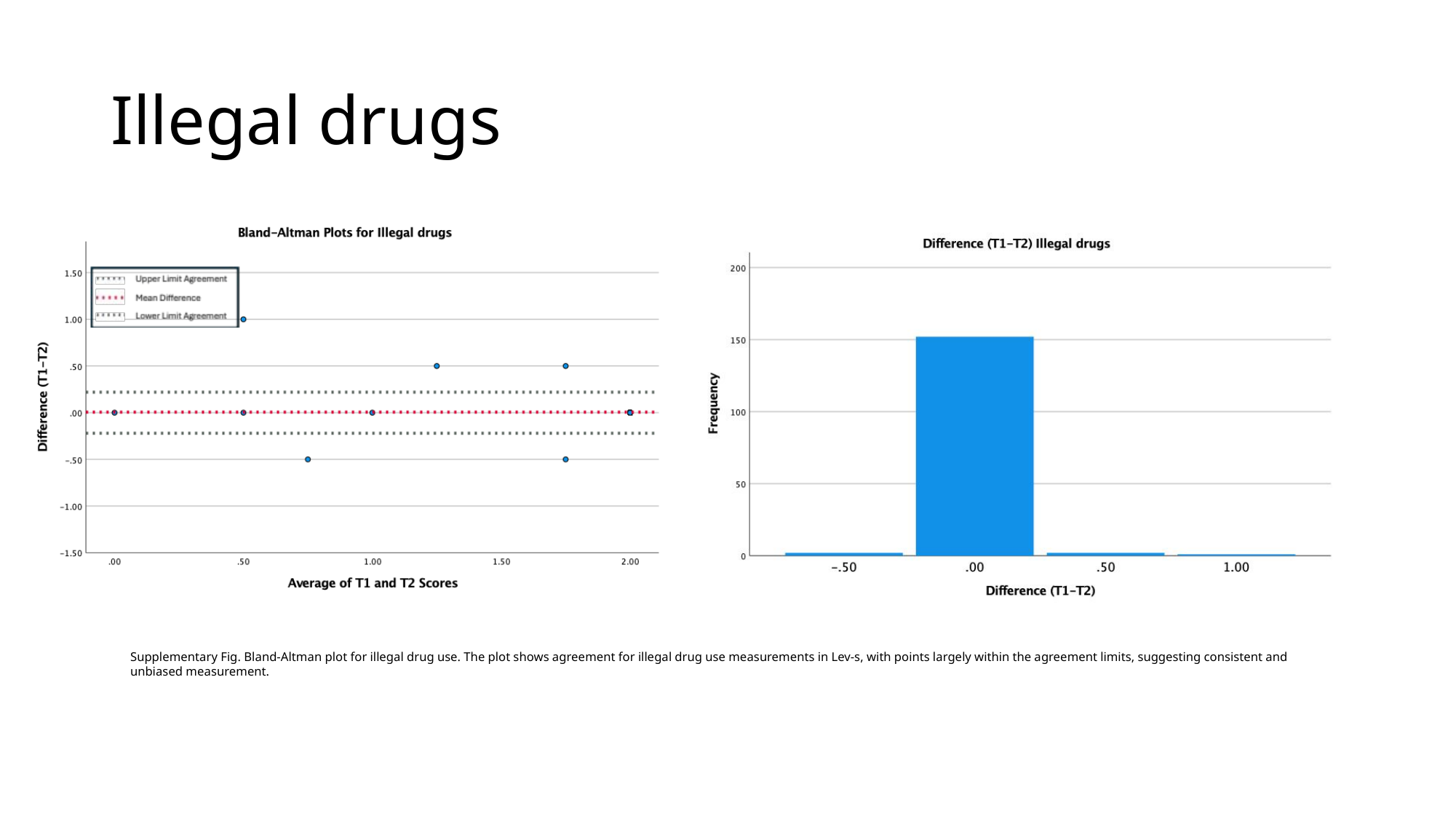

# Illegal drugs
Supplementary Fig. Bland-Altman plot for illegal drug use. The plot shows agreement for illegal drug use measurements in Lev-s, with points largely within the agreement limits, suggesting consistent and unbiased measurement.

## Slide 8
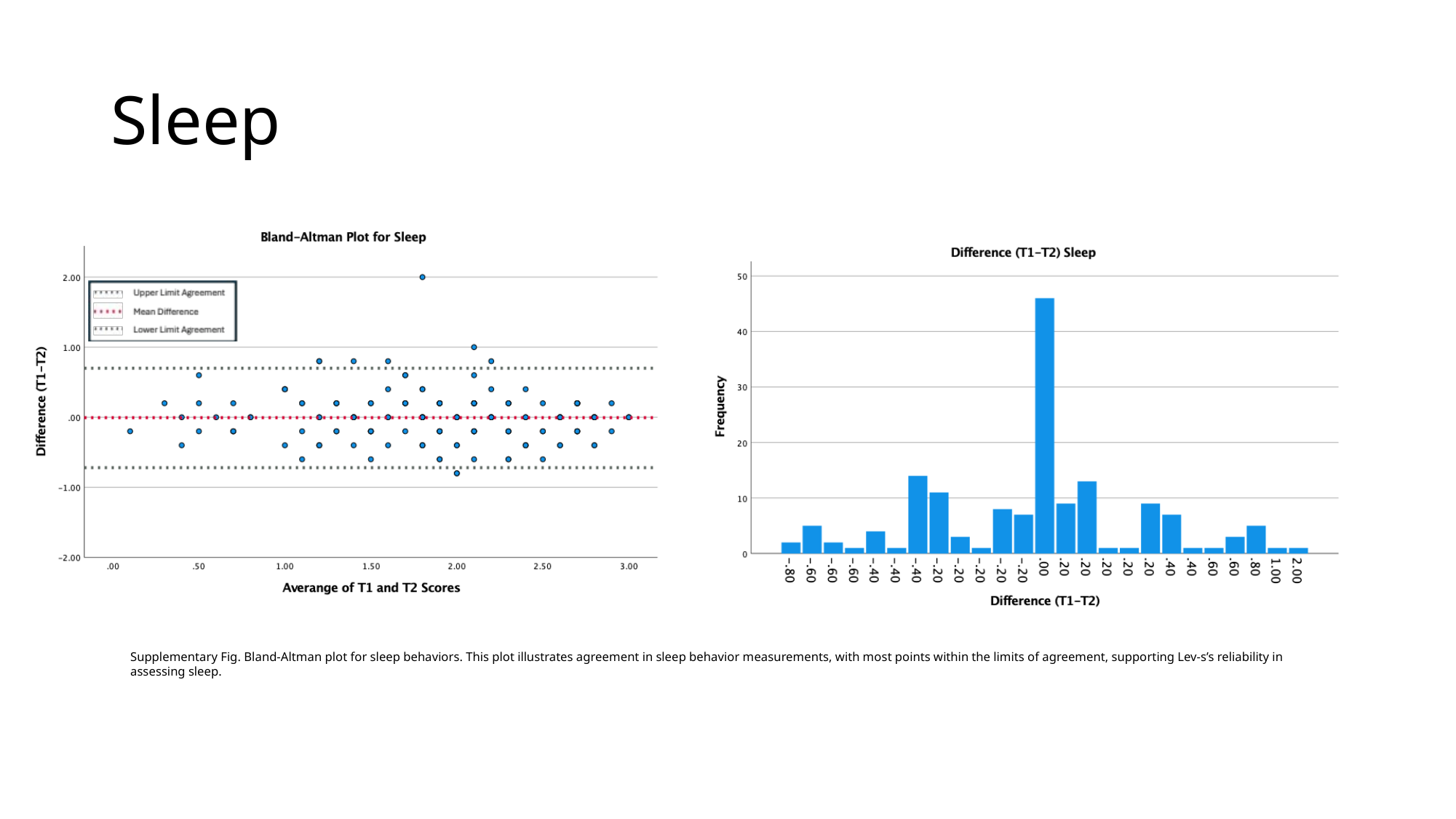

# Sleep
Supplementary Fig. Bland-Altman plot for sleep behaviors. This plot illustrates agreement in sleep behavior measurements, with most points within the limits of agreement, supporting Lev-s’s reliability in assessing sleep.

## Slide 9
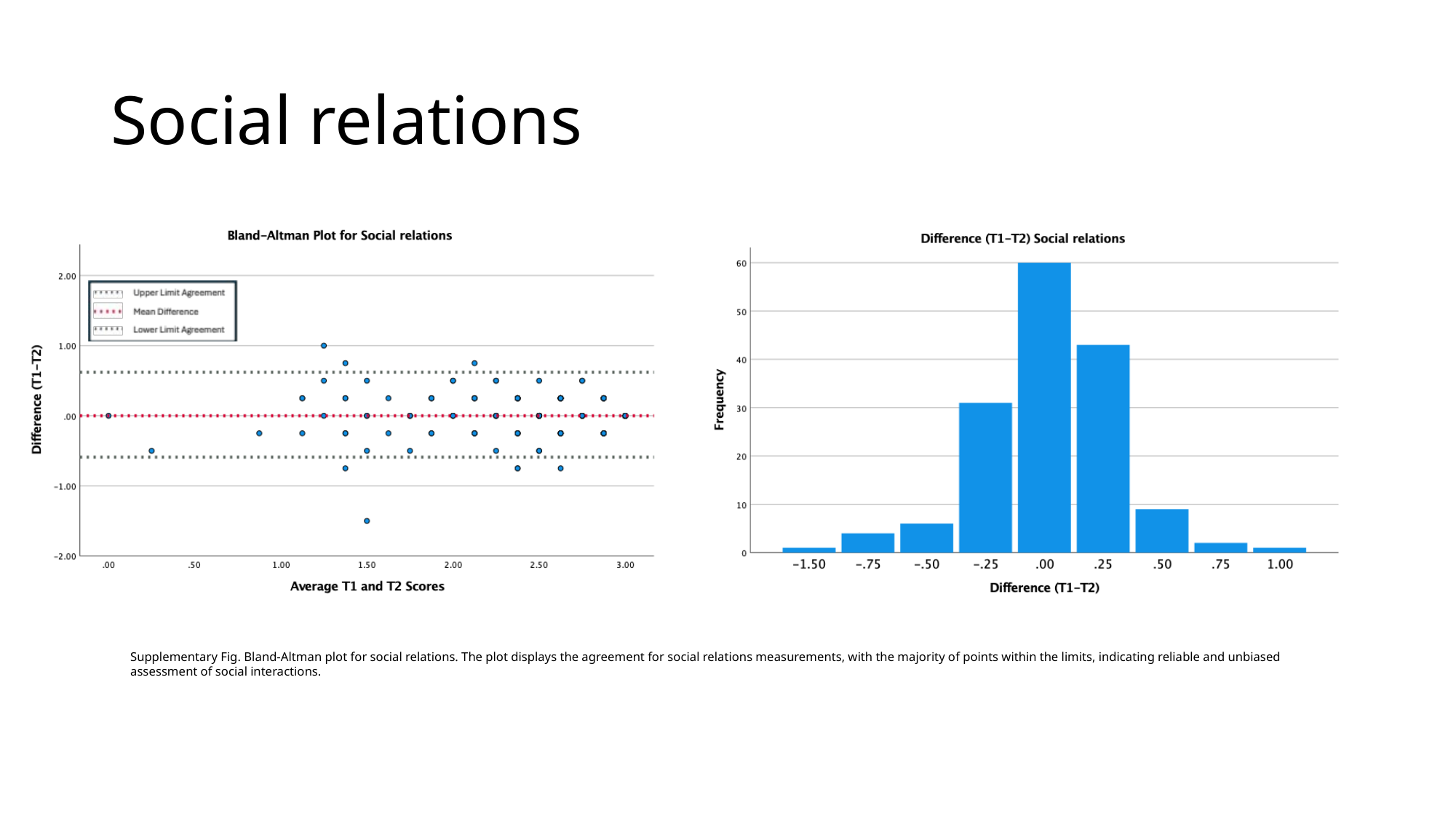

# Social relations
Supplementary Fig. Bland-Altman plot for social relations. The plot displays the agreement for social relations measurements, with the majority of points within the limits, indicating reliable and unbiased assessment of social interactions.

## Slide 10
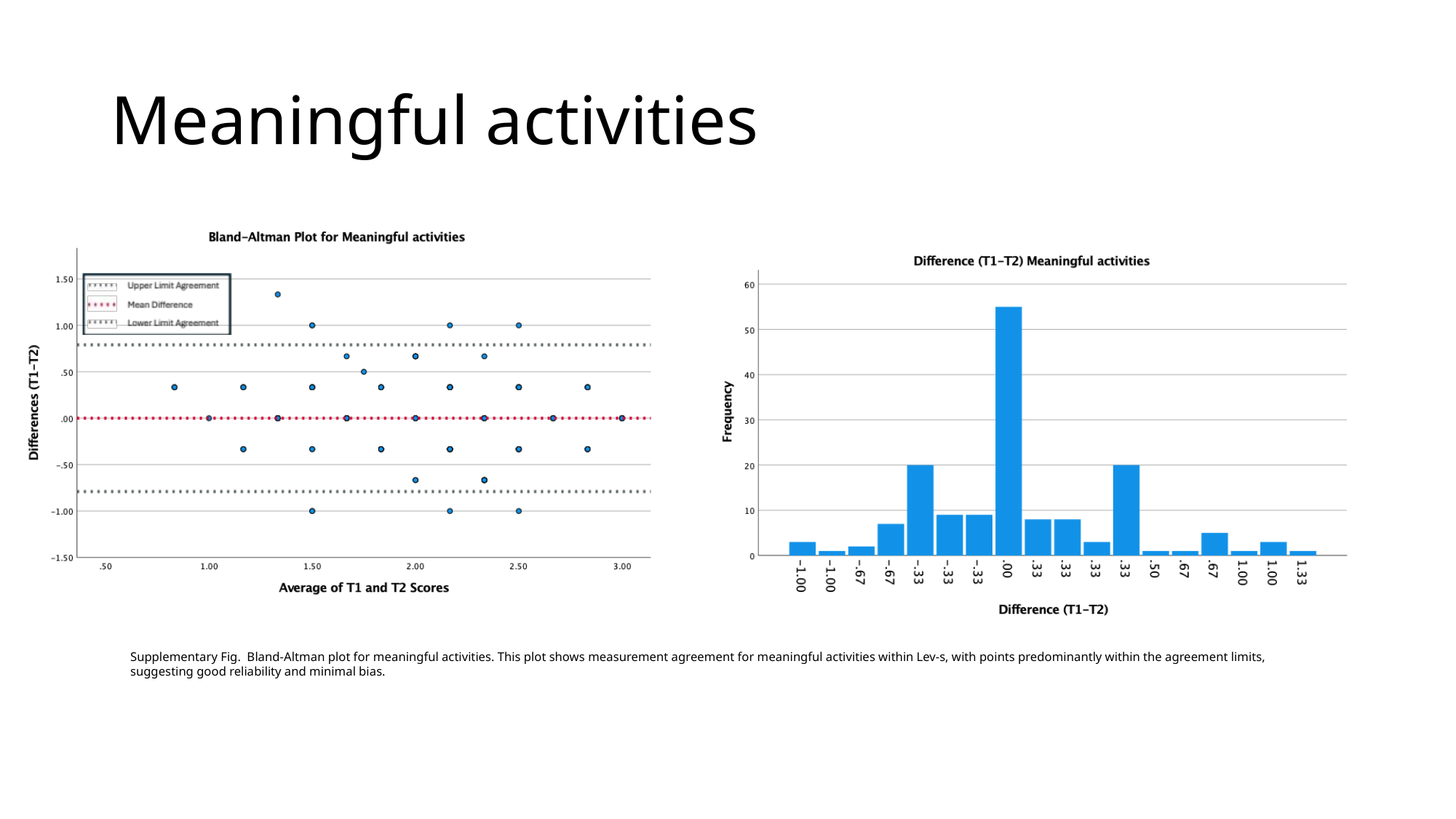

# Meaningful activities
Supplementary Fig. Bland-Altman plot for meaningful activities. This plot shows measurement agreement for meaningful activities within Lev-s, with points predominantly within the agreement limits, suggesting good reliability and minimal bias.

## Slide 11
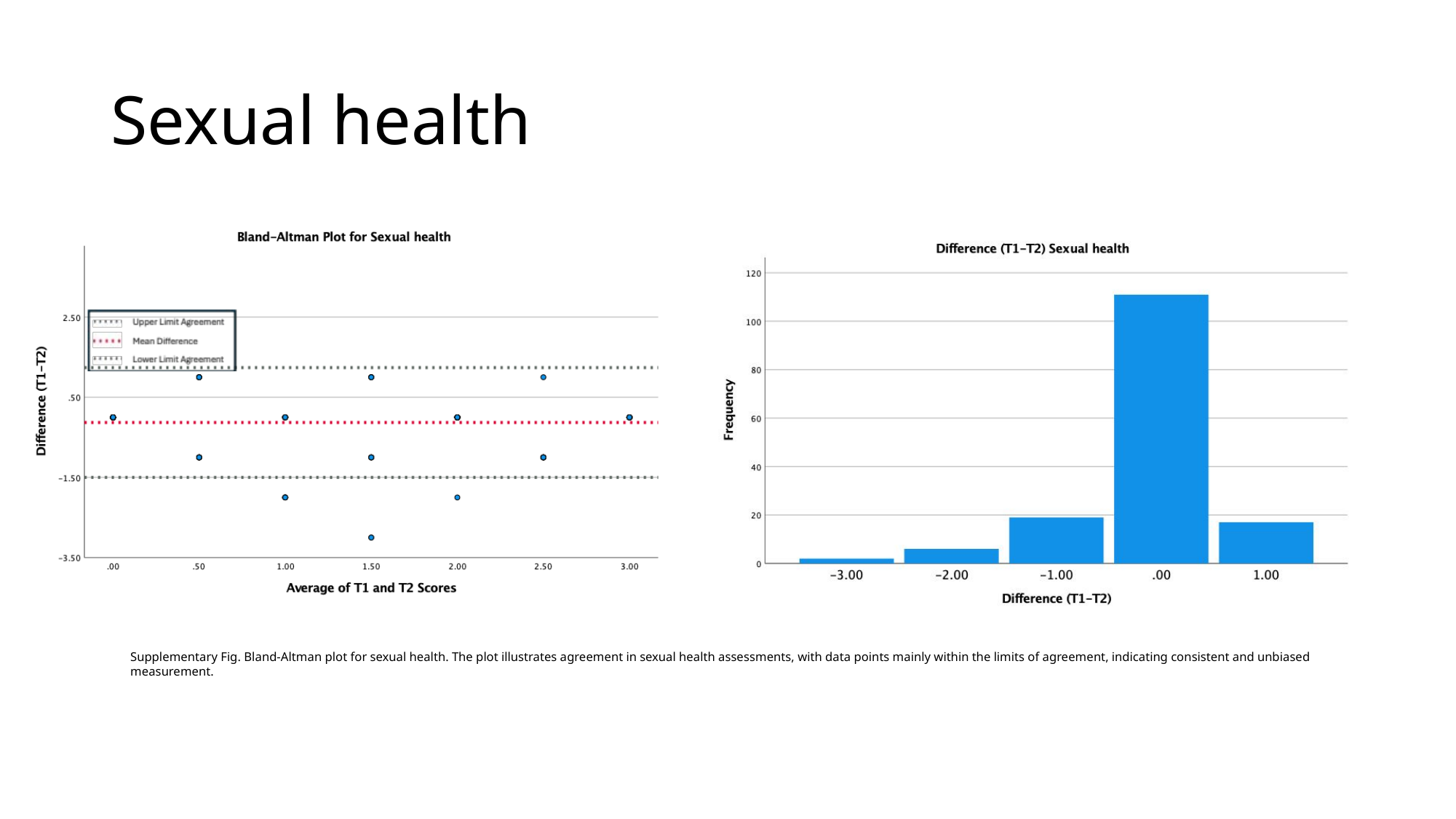

# Sexual health
Supplementary Fig. Bland-Altman plot for sexual health. The plot illustrates agreement in sexual health assessments, with data points mainly within the limits of agreement, indicating consistent and unbiased measurement.

## Slide 12
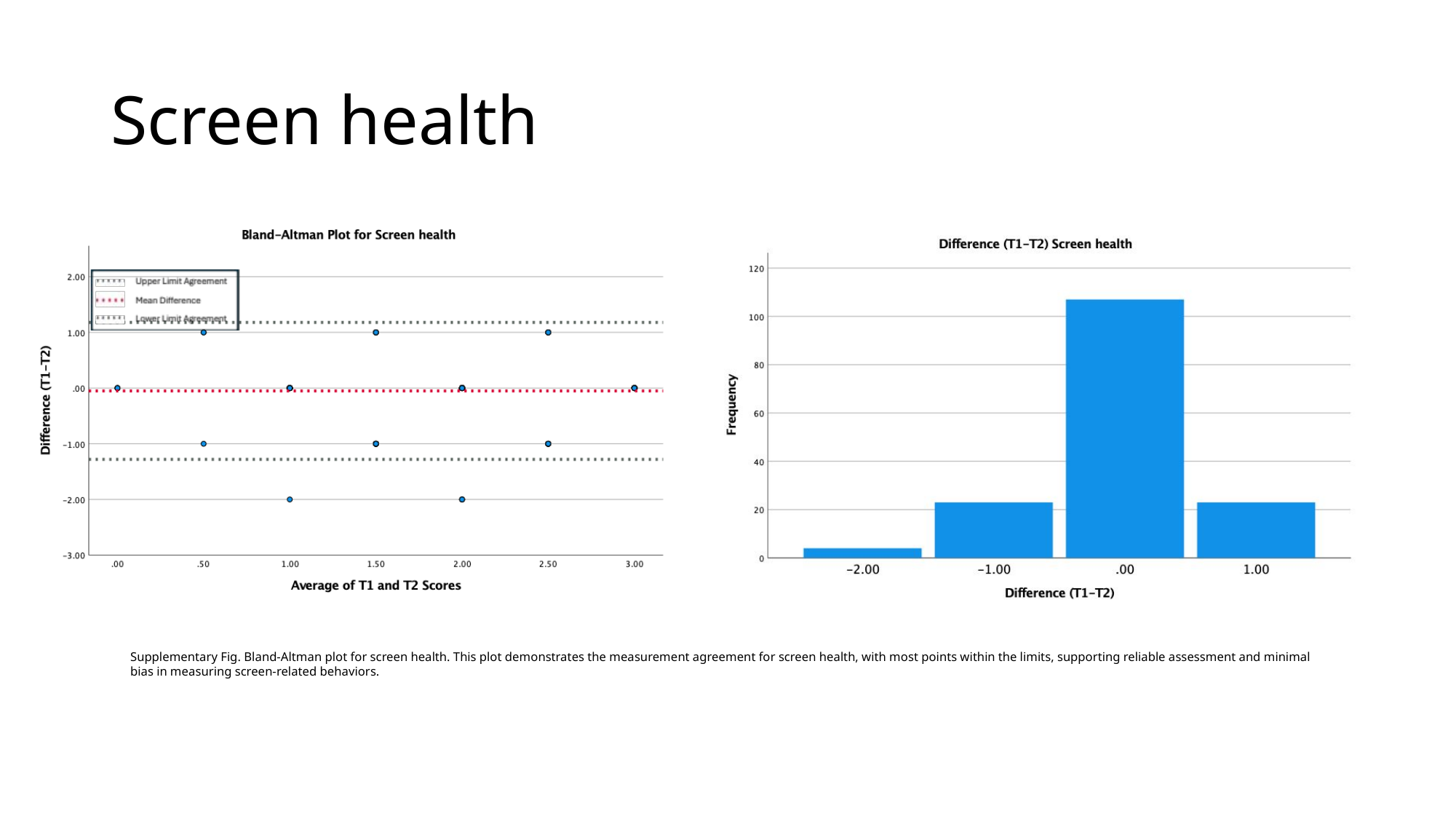

# Screen health
Supplementary Fig. Bland-Altman plot for screen health. This plot demonstrates the measurement agreement for screen health, with most points within the limits, supporting reliable assessment and minimal bias in measuring screen-related behaviors.
